# VEGFR1 signaling in retinal angiogenesis and microinflammation

**DOI:** 10.1016/j.preteyeres.2021.100954

**Published:** 2021-02-25

**Authors:** Akiyoshi Uemura, Marcus Fruttiger, Patricia A. D’Amore, Sandro De Falco, Antonia M. Joussen, Florian Sennlaub, Lynne R. Brunck, Kristian T. Johnson, George N. Lambrou, Kay D. Rittenhouse, Thomas Langmann

**Affiliations:** a Department of Retinal Vascular Biology, Nagoya City University Graduate School of Medical Sciences, 1 Kawasumi Mizuho-cho, Mizuho-ku, Nagoya, 467-8601, Japan; b UCL Institute of Ophthalmology, University College London, 11-43 Bath Street, London, EC1V 9EL, UK; c Schepens Eye Research Institute of Massachusetts Eye and Ear, 20 Staniford Street, Boston, MA, 02114, USA; d Angiogenesis Laboratory, Institute of Genetics and Biophysics “Adriano Buzzati-Traverso”, Via Pietro Castellino 111, 80131 Naples, Italy; ANBITION S.r.l., Via Manzoni 1, 80123, Naples, Italy; e Department of Ophthalmology, Charité–Universitätsmedizin Berlin, Hindenburgdamm 30, 12200 Berlin, and Augustenburger Platz 1, 13353, Berlin, Germany; f Sorbonne Université, INSERM, CNRS, Institut de la Vision, 17 rue Moreau, F-75012, Paris, France; g Bayer Consumer Care AG, Pharmaceuticals, Peter-Merian-Strasse 84, CH-4052 Basel, Switzerland; h Laboratory for Experimental Immunology of the Eye, Department of Ophthalmology, Faculty of Medicine and University Hospital Cologne, University of Cologne, Joseph-Stelzmann-Str. 9, 50931, Cologne, Germany

**Keywords:** Angiogenesis, Microinflammation, Placental growth factor (PlGF), Vascular endothelial growth factor-A (VEGF-A), Vascular endothelial growth factor receptor 1 (VEGFR1)

## Abstract

Five vascular endothelial growth factor receptor (VEGFR) ligands (VEGF-A, -B, –C, -D, and placental growth factor [PlGF]) constitute the VEGF family. VEGF-A binds VEGF receptors 1 and 2 (VEGFR1/2), whereas VEGF-B and PlGF only bind VEGFR1. Although much research has been conducted on VEGFR2 to elucidate its key role in retinal diseases, recent efforts have shown the importance and involvement of VEGFR1 and its family of ligands in angiogenesis, vascular permeability, and microinflammatory cascades within the retina. Expression of VEGFR1 depends on the microenvironment, is differentially regulated under hypoxic and inflammatory conditions, and it has been detected in retinal and choroidal endothelial cells, pericytes, retinal and choroidal mononuclear phagocytes (including microglia), Müller cells, photoreceptor cells, and the retinal pigment epithelium. Whilst the VEGF-A decoy function of VEGFR1 is well established, consequences of its direct signaling are less clear. VEGFR1 activation can affect vascular permeability and induce macrophage and microglia production of proinflammatory and proangiogenic mediators. However the ability of the VEGFR1 ligands (VEGF-A, PlGF, and VEGF-B) to compete against each other for receptor binding and to heterodimerize complicates our understanding of the relative contribution of VEGFR1 signaling alone toward the pathologic processes seen in diabetic retinopathy, retinal vascular occlusions, retinopathy of prematurity, and age-related macular degeneration. Clinically, anti-VEGF drugs have proven transformational in these pathologies and their impact on modulation of VEGFR1 signaling is still an opportunity-rich field for further research.

## 1. Introduction

The retina is nourished by two distinct vascular networks: the inner retina is maintained by the retinal vasculature, whereas the outer retina depends on the choroidal vasculature. The retinal vasculature comprises tightly sealed endothelial cells (ECs) surrounded by pericytes and glial cells, forming the inner blood–retina barrier (BRB) (Díaz-Coránguez et al., 2017; Klaassen et al., 2013). In contrast, the choroidal vasculature is highly permeable, consisting of fenestrated ECs with fewer pericytes, from which oxygen diffuses through the monolayered retinal pigment epithelial (RPE) cells of the outer BRB to reach photoreceptor cells (Fields et al., 2020; Kur et al., 2012). At both the inner and outer barriers, pathological changes can lead to uncontrolled formation of new fragile blood vessels and extravasation in both the retinal and choroidal vascular beds, which can lead to severe vision impairment or blindness (Klaassen et al., 2013).

The most common disease affecting the retinal vasculature is diabetic retinopathy (DR), the prevalence of which increases with duration of diabetes (approximately 20% versus 75% in individuals with diabetes for <10 versus ≥20 years) and levels of glycosylated hemoglobin (approximately 20% versus 50% in individuals with levels ≤7.0 versus >9.0%), and is higher in those with type 1 versus type 2 (approximately 75% versus 25%) diabetes (Yau et al., 2012). Hyperglycemia in diabetes is the major long-term determinant of vascular changes in DR (Klaassen et al., 2013); damage to the ECs and pericytes of the inner BRB contribute to subsequent retinal edema and hemorrhage within the retina and impaired vision (Kempen et al., 2004). In proliferative DR (PDR), an advanced form of DR, retinal microvascular alterations lead to tissue ischemia and retinal neo-angiogenesis, which are often accompanied by development of contractile fibrovascular membranes.

The main pathology that occurs in the outer retina is age-related macular degeneration (AMD). As AMD progresses, sustained stress to RPE cells leads to a loss of photoreceptor cells, RPE cells, and the underlying choriocapillaris, which in the late stage of “dry AMD” manifests as geographic atrophy (GA) and leads to vision loss. In “wet AMD”, choroidal neovascularization (CNV) causes a wide variety of anatomical disruptions in the neural architecture of the macula, such as retinal edema and detachment with hemorrhagic exudates, as well as subretinal fibrosis. Again, these changes lead to vision impairment and, in more extreme cases, vision loss. Although vascular changes are the hallmark of DR and AMD, there is also an increasing appreciation that within both DR and AMD there is chronic microinflammation that occurs at a cellular level in the absence of tissue injury or infection and is also referred to as “subclinical” or “low-grade” inflammation (Antonelli and Kushner, 2017), “para-inflammation” (Chen and Xu, 2015; Xu et al., 2009), or “inflamm-aging” (Subhi et al., 2019).

Vascular endothelial growth factor (VEGF) is viewed as a pivotal mediator of pathology in both AMD and DR and is a target of current therapeutic interventions (Aiello, 2008). However, the VEGF signaling pathway is complex and includes multiple ligand–receptor interactions that regulate diverse processes in different cell types, in a context-dependent manner (Penn et al., 2008). To date, the best characterized process is activation of VEGF receptor 2 (VEGFR2) tyrosine kinase (TK) in ECs by VEGF-A, which induces angiogenesis and increases vascular permeability (Peach et al., 2018). In contrast, VEGF receptor 1 (VEGFR1) functions, in part, as a decoy for VEGF-A, attenuating VEGFR2/VEGF-A–mediated outcomes (Koch and Claesson-Welsh, 2012). In addition, an established body of evidence indicates disease-specific roles of direct VEGFR1 signaling, which are independent of VEGFR1 decoy activity, in particular in immune cells expressing VEGFR1 (Clauss et al., 1996; Crespo-Garcia et al., 2017; Huang et al., 2013; Luttun et al., 2002; Muramatsu et al., 2010). Yet the complexities in the ligand–receptor interactions and their differential expression have so far precluded a clear understanding of VEGFR1 functions.

In this review, we summarize the current understanding of the signals mediated by VEGFR1 and its ligands – VEGF-A, VEGF-B, and placental growth factor (PlGF) – within the retinal microenvironment in both healthy and pathologic states. We further discuss the possibility of translating the knowledge gained from basic science into the clinical management of DR and AMD, as well as other eye diseases. The goal of this review is to highlight the key research in this area and guide future research in this constantly evolving field. We recognize that this area of research is extensive, spanning pharmacology to animal models to clinical trials and it is clear that the science of VEGFR1 continues to grow, as evidenced by the consistently increasing number of publications on this topic. Accordingly, the review begins by introducing the current biology of the VEGFR1 itself and in context with VEGFR2, followed by an overview of disease-driven biological processes where VEGFR1 is known to have a role, focusing on retinal vascular and macular degenerative diseases.

## 2. Molecular signaling mechanisms in the VEGF family

### 2.1. Basic VEGF receptor properties

There are three evolutionarily related VEGF receptors in humans: VEGFR1 (FLT1), VEGFR2 (KDR/FLK1), and VEGFR3 (FLT4) (Grassot et al., 2006). They all comprise an extracellular, ligand-binding domain composed of seven immunoglobulin-like loops, a transmembrane domain, a juxtamembrane domain, a split intracellular TK domain, and a C-terminal tail; all three have different ligand-binding properties and biological functions (D’Amore, 1994; De Falco, 2012; de Vries et al., 1992; Ebos et al., 2004; Jeltsch et al., 2013; Shibuya, 2013; Takahashi and Shibuya, 1997; Terman et al., 1992; Vaisman et al., 1990).

There are five VEGFR ligands (VEGF-A, -B, –C, -D, and PlGF) collectively known as the VEGF family. Isolated and cloned in 1989, VEGF-A was confirmed as a disulfide-linked dimeric glycoprotein with EC growth-promoting properties (Ferrara, 2011; Jakeman et al., 1992; Keck et al., 1989; Leung et al., 1989). Of all the VEGF family members, PlGF shares the greatest homology with VEGF-A, despite its unique nomenclature, which derives from the fact that it was initially isolated from a human placental complementary DNA library (Iyer et al., 2001). The VEGF-A mRNA contains eight exons, the splicing of which gives rise to a variety of isoforms. To date, 16 distinct VEGF-A isoforms have been identified most commonly from six transcripts: VEGF_111_, VEGF_121_, VEGF_145_, VEGF_165_, VEGF_189_, and VEGF_206_; the subscripted numbers denoting the number of amino acids present (Peach et al., 2018). The isoforms have different affinities for extracellular matrix components, VEGF receptors and associated coreceptors, and the expression profile of each isoform varies among tissues (Ng et al., 2001). One of the primary differences among the VEGF-A isoforms is their ability to bind heparan sulfate proteoglycan, affecting their diffusibility within tissues: the larger isoforms can bind heparan sulfate proteoglycan, whereas VEGF-A_120_ in mice and VEGF-A_121_ in humans do not (Bridgett et al., 2017; Stalmans et al., 2002). Alternative splicing of the VEGF-A pre-mRNA may also give rise to a non- or anti-angiogenic family of isoforms (VEGF-A_xxxb_) as well as the pro-angiogenic family (VEGF-A_xxxa_) (Bowler and Oltean, 2019; Peach et al., 2018). However, the relevance of these transcripts has been contested (Bridgett et al., 2017; Harris et al., 2012; Lomet et al., 2018). Furthermore, alternative splicing also gives rise to multiple isoforms of PlGF and VEGF-B (Olofsson et al., 1996; Yang et al., 2003).

### 2.2. Modulation of VEGFR1/2 signaling

VEGF-A binds VEGFR1 and VEGFR2, whereas VEGF-B and PlGF only bind VEGFR1, and VEGF-C and VEGF-D primarily bind VEGFR3 (Jeltsch et al., 2013), although proteolytically processed VEGF-C and VEGF-D can also bind VEGFR2 (Achen et al., 1998; Joukov et al., 1996, 1997; Masoumi Moghaddam et al., 2012; Stacker et al., 1999). However, as the VEGF-C/VEGF-D/VEGFR3 pathway mainly regulates the formation of lymphatic vessels, which are absent in the retina (Alitalo, 2002), this review focuses primarily on VEGFR1 and its ligands (VEGF-A, VEGF-B, and PlGF), and delineates the roles between the VEGFR1 and VEGFR2 receptors in retinal development, homeostasis, and disease.

VEGFR1/2 activation and signaling are heavily influenced by a number of elements, and a key one is the availability of the VEGFR ligands themselves, which are modulated by a large array of mechanisms. First, the availability of VEGFR ligands depends on transcriptional and post-transcriptional regulation of their expression. Second, specific ligands can bind and activate VEGFR1, causing it to signal via its own kinase domain, but VEGFR1 also has indirect effects on VEGFR2 activity by acting as a decoy receptor for VEGFR2 ligands (Koch and Claesson-Welsh, 2012). Third, VEGF-A, VEGF-B, and PlGF can compete for VEGFRs and extracellular matrix binding sites (Koch and Claesson-Welsh, 2012). Fourth, VEGF-A, VEGF-B, and PlGF can form heterodimers and VEGFR subtypes can form homodimers or heterodimers, depending on the binding ligand (Koch and Claesson-Welsh, 2012). Fifth, VEGFR1/2 activity can also be modulated by neuropilins and the glycocalyx component, endomucin, which act as VEGF co-receptors (Alvarez-Aznar et al., 2017; LeBlanc et al., 2019).

#### 2.2.1. Expression of VEGFR1 ligands and VEGFRs

**Fig. 1.**
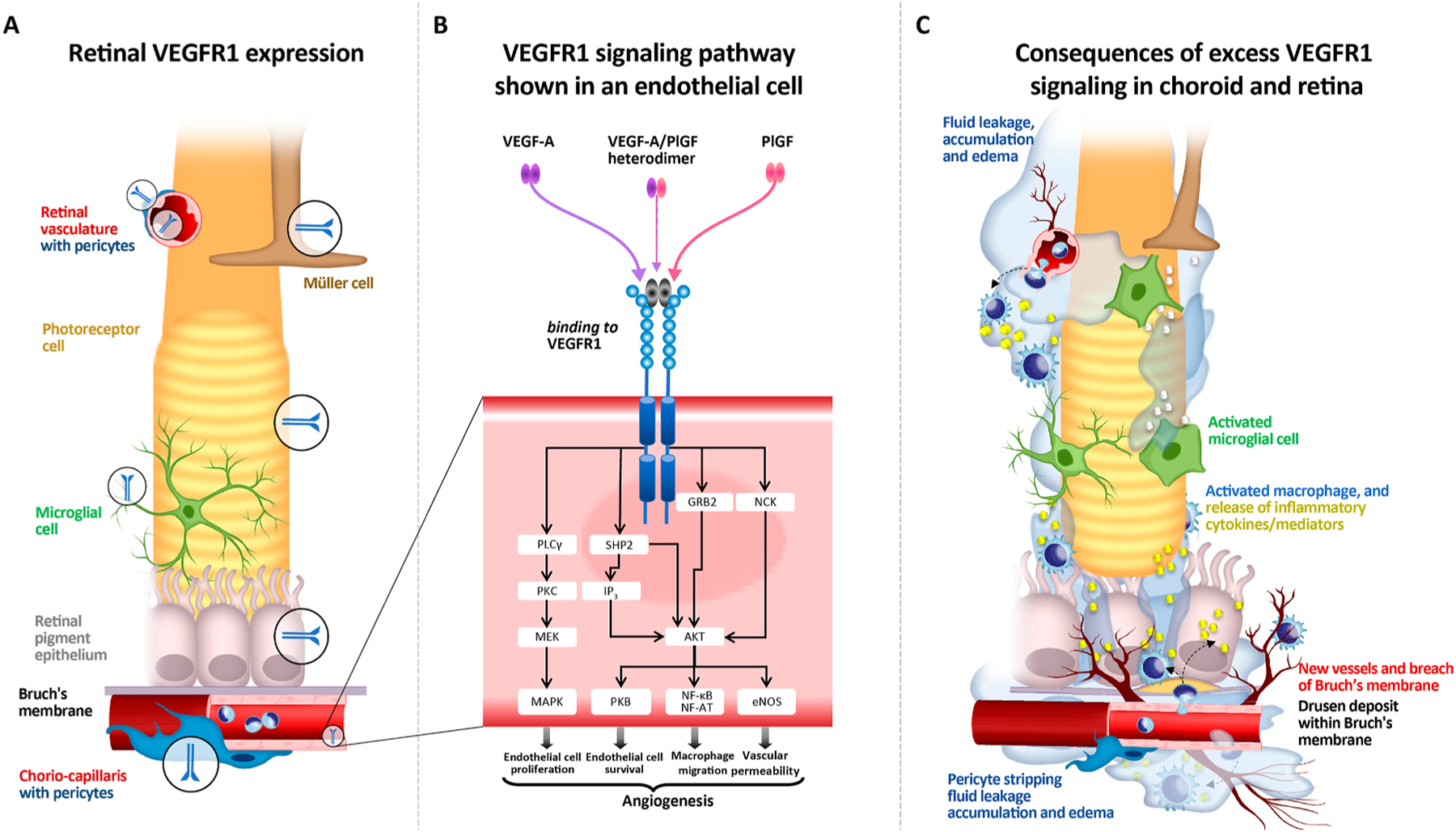
Schematic representation of VEGFR1 in the choroid and retina and VEGFR1 signaling (for illustrative purposes and not to scale). **A.** VEGFR1 expression in various types of cells, including vascular endothelial cells, pericytes, mononuclear phagocytes, Müller cells, photoreceptor cells, and the retinal pigment epithelium. **B.** VEGFR1 signaling through VEGF-A and/or PlGF, via a variety of different pathways, contributing to numerous pathologic processes in endothelial cells and pericytes in the choroid and retina: pericyte ablation, loss of tight junctions between endothelial cells, vasodilation, breakdown of the blood-retinal-barrier, increased permeability and leakage, edema and hemorrhage in surrounding tissue, neutrophil migration and monocyte migration and differentiation into macrophages, influx of pro-inflammatory cytokines e.g. tumor necrosis factor-α and interleukin-6 into surrounding tissue, increased angiogenic sprouts and neoangiogenesis. **C.** Consequences of excess VEGFR1 signaling in the choroid and retina: in retinal pigment cells: neoangiogenesis of vessels through Bruch’s membrane into the retinal pigment epithelium, loss of retinal pigment cells; in photoreceptor cells: loss of photoreceptor integrity, rod death and cone segment loss; in Müller cells: Müller cell activation; in microglial cells: recruitment, accumulation, and activation of microglial cells and other retinal macrophages, release of pro-inflammatory cytokines e.g. platelet-derived growth factor-A, soluble intracellular adhesion molecule-1, CC chemokine ligand 2, and interleukin-8, leading to the development of hyperreflective foci.

VEGFR1 expression has been detected in various types of cells, including retinal and choroidal ECs (Cao et al., 2010; Fruttiger, 2002; Stewart et al., 2011), retinal pericytes (Cao et al., 2010; Eilken et al., 2017), retinal and choroidal mononuclear phagocytes (Couturier et al., 2014; Huang et al., 2013; Ogura et al., 2017), Müller cells (Stitt et al., 1998), photoreceptor cells (Luo et al., 2013), and RPE cells (Luo et al., 2013) as shown in Fig. 1a. It should be noted that the VEGFR1 expression in these cell types is variable depending on their microenvironments in developmental, homeostatic, and disease conditions. In particular, oxygen concentration and inflammation largely influence the expression levels of VEGFR1 as well as its ligands (Hata et al., 1995; Philipp et al., 2000).

Michaelson et al. first introduced the concept that damage to the ECs and/or pericytes of the vascular network may lead to vessel closure and ultimately, hypoxia within the retina (Michaelson et al., 1954). Soon afterwards, Wise proposed the presence of a “hypoxia-induced growth factor” (Wise, 1961), which was identified as VEGF-A over the subsequent decades. Mechanistically, it has been shown that tissue exposure to hypoxia provokes an adaptive response that is reliant on the ability of retinal cells to detect alterations in intracellular oxygen tension.

Hypoxia-induced expression of VEGF-A can be mediated by both transcriptional and post-transcriptional mechanisms. The transcriptional response depends on hypoxia-inducible factor (HIF)-1 and HIF-2, heterodimeric transcription factors that modulate the expression of a large set of genes through binding to the hypoxia-responsive element located in the promoters or other genomic regulatory regions (Forsythe et al., 1996; Gerber et al., 1997; Maxwell and Ratcliffe, 2002; Semenza, 2001). VEGF-A mRNA is intrinsically labile and contains destabilizing elements in its 5′, 3’ untranslated, and coding regions (Dibbens et al., 1999; Levy, 1998; Yao et al., 2013). Hypoxic conditions stabilize VEGF-A mRNA (Dibbens et al., 1999; Levy, 1998; Yao et al., 2013), increasing its half-life from about 45 min to more than 8 h (Shima et al., 1995). This post-transcriptional mechanism can have a greater impact on VEGF-A protein production than transcriptional regulation.

HIF-mediated gene regulation also impacts expression of other members of the VEGF family and related receptors. For instance, HIF-1 activates transcription of VEGFR1, but not VEGFR2 (Gerber et al., 1997). More recently, the molecular mechanisms underlying the positive modulation of PlGF expression by hypoxia in vascular cells at transcriptional (Tudisco et al., 2017) and post-transcriptional (Xiang et al., 2014) levels have been unraveled. Furthermore, hypoxia-related modulation of PlGF expression has been shown to be mediated by metal-responsive transcription factor-1 in immortalized/H-Ras–transformed mouse embryonic fibroblasts (Green et al., 2001), and by nuclear factor-κB in human embryonic kidney 293 cells (Cramer et al., 2005).

Several other mechanisms regulate gene expression in the VEGF family. For instance, VEGF-A expression is upregulated by several growth factors, including epidermal growth factor, transforming growth factors (TGF) α and β, insulin-like growth factor-1, fibroblast growth factor, and platelet-derived growth factor (PDGF) (Ferrara et al., 2003). Inflammatory cytokines, such as interleukin (IL)-1β and IL-6, stimulate expression of VEGF-A in several cell types, modulating angiogenesis and vascular permeability in inflammatory conditions (Ferrara et al., 2003). Oncogenic mutations can also influence VEGF-A expression (Ferrara et al., 2003). Similarly, activation of oncogenes (Ras), inflammatory cytokines (IL-1β and tumor necrosis factor [TNF]-α), and several growth factors can also positively modulate PlGF expression in pathologic conditions, including many types of cancer and chronic inflammatory conditions (Kim et al., 2012).

#### 2.2.2. Decoy function of VEGFR1

Animal studies provided evidence of the essential function of VEGFR1 in vascular development as mice lacking VEGFR1 die at embryonic day 8.5 due to an excess of ECs, which assemble into disorganized tubules (Fong et al., 1995). In contrast, mice engineered to express a truncated, non-signaling form of VEGFR1 lacking the TK domain (*VEGFR1-TK*−/−) (Hiratsuka et al., 1998) are healthy and fertile with close to normal vascularization. Similarly, knock-out (KO) mice for genes encoding the VEGFR1-specific ligands, PlGF (Apicella et al., 2018; Carmeliet et al., 2001) and VEGF-B (Bellomo et al., 2000), are also largely normal.

Although VEGF-A binds VEGFR1 with higher affinity than VEGFR2 (K_d_ = 15 pM vs 750 pM), VEGFR1 exhibits 10-times lower TK activity (Sawano et al., 2001; Shinkai et al., 1998). Based on initial observations, it appears that one role of VEGFR1 is to act as a decoy receptor for VEGF-A rather than a signaling mediator, which limits the activity of the VEGF-A/VEGFR2 axis, at least in physiological settings (Carmeliet et al., 2001). In addition, a soluble/secreted version of VEGFR1 (sVEGFR1) can be produced via alternative splicing or proteolytic cleavage retaining the extracellular ligand–binding domains of VEGFR1 (Kendall and Thomas, 1993; Raikwar et al., 2013), lowering the availability of free VEGF-A in the extracellular space and indirectly modulating the intensity of VEGFR2 signaling (Kappas et al., 2008). The powerful anti-VEGF-A activity of sVEGFR1 is seen in the cornea, where it is strongly expressed and plays a crucial role in maintaining corneal avascularity (Ambati et al., 2006).

#### 2.2.3. Competition among VEGFR1 ligands

Because VEGFR1 functions as a decoy receptor for VEGF-A, the other two VEGFR1 ligands, PlGF and VEGF-B, can indirectly affect VEGF-A availability by competing for VEGFR1 binding. For example, increased levels of VEGF-B can increase unbound VEGF-A levels by preventing VEGF-A from being trapped by VEGFR1, indirectly leading to increased VEGFR2 activation (Anisimov et al., 2013; Kivela et al., 2014; Robciuc et al., 2016). In other words, despite its lack of affinity for VEGFR2, VEGF-B can still indirectly activate VEGFR2 signaling, if VEGF-A is present.

Likewise, PlGF can also increase unbound VEGF-A levels by competing for VEGFR1 binding (Yang et al., 2013) and displacing VEGF-A from VEGFR1 (Carmeliet et al., 2001; Kowalczuk et al., 2011; Park et al., 1994). However, competition between PlGF and VEGF-A for VEGFR1 binding is complicated by PlGF also being capable of directly activating VEGFR1 signaling. Indeed, PlGF can directly stimulate vessel growth by inducing proliferation, migration, and survival of ECs (Adini et al., 2002; Carmeliet et al., 2001; Ziche et al., 1997a), as well as vessel maturation, by increasing the proliferation and recruitment of vascular smooth muscle cells (Bellik et al., 2005; Yonekura et al., 1999). Thus, when interpreting biological outcomes, it is important to consider the possibility of effects of PlGF and VEGF-B acting directly via VEGFR1, or indirectly via VEGFR2 due to VEGF-A displacement.

In summary, PlGF and VEGF-B are able to compete with VEGF-A in binding to VEGFR1, freeing up VEGF-A. The relative binding affinities of VEGF-A and PlGF to VEGFR1 have been assessed under various conditions, including levels of glycosylation of the ligands and binding to specific domains of the receptor (Huang et al., 2019a; Jiao et al., 2019). To date, it remains unclear whether VEGF-A or PlGF binds more tightly to VEGFR1.

#### 2.2.4. Heterodimerization of VEGFR1 ligands and VEGFRs

**Fig. 2.**
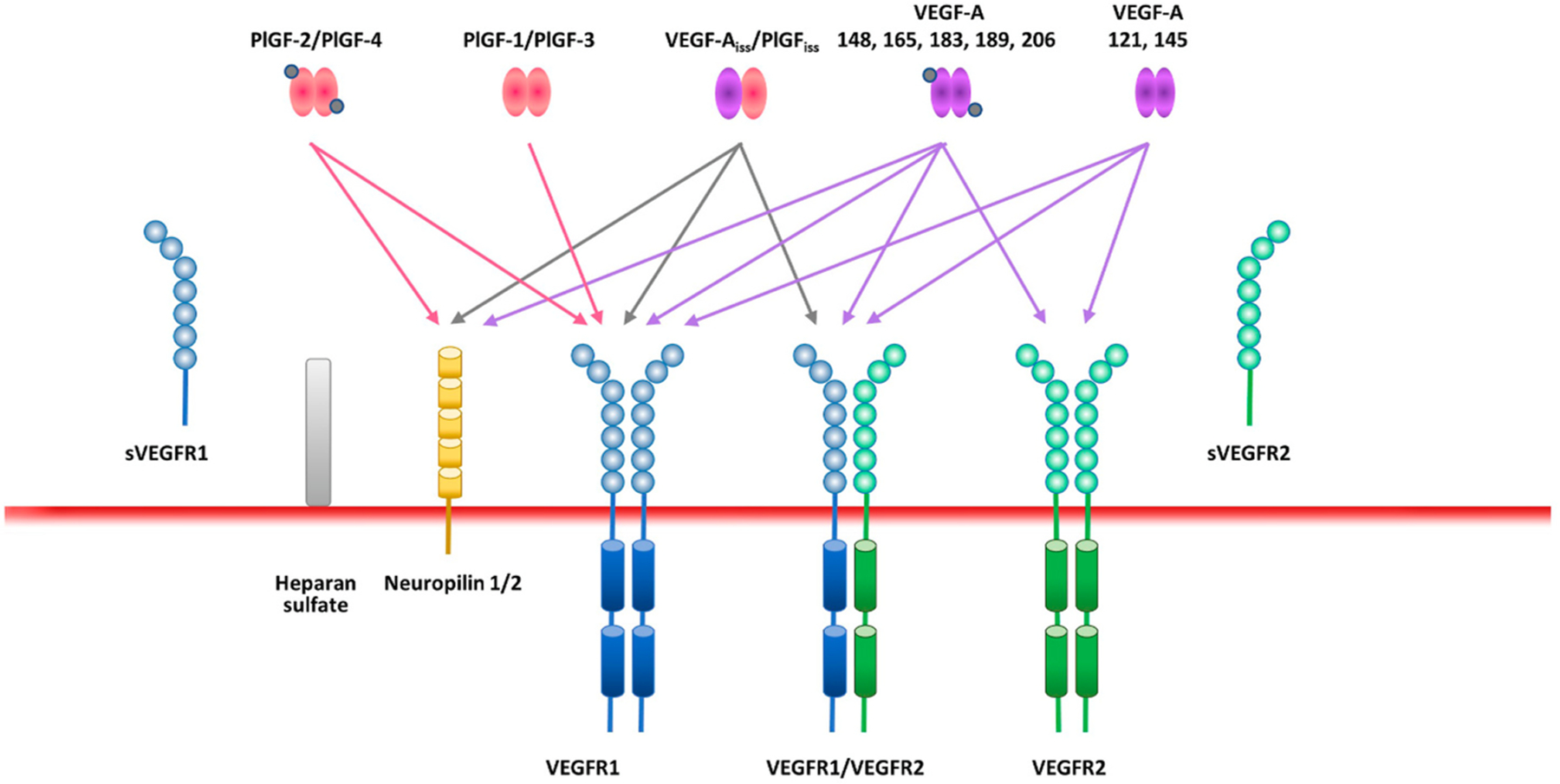
VEGFR1 and VEGFR2 and the family of ligands and co-receptors. There are five VEGFR ligands, of which VEGF-A binds to both VEGFR1 and VEGFR2, and PlGF only binds VEGFR1. Splicing creates isoforms of both VEGF-A and PlGF. In addition, soluble/secreted versions of VEGFR1 and VEGFR2 can be produced via alternative splicing or proteolytic cleavage retaining the extracellular ligand–binding domains. Furthermore, VEGF-A and PlGF are able to bind neuropilin (NRP) 1 and 2, bridging VEGFRs and NRP1 or NRP2 to create holoreceptor complexes. VEGF-A and PlGF ligands and the VEGFR1 and VEGFR2 receptors can form heterodimers as well as homodimers. Functional synergistic effects of PlGF and VEGF-A are due to sharing of the common receptor, VEGFR1, and the ability to heterodimerize.

VEGF-A, VEGF-B, and PlGF can form heterodimers (Fig. 2) if they are co-expressed in the same cell (Cao et al., 1996; DiSalvo et al., 1995). The formation of VEGF-A/PlGF heterodimers can reduce the number of VEGF-A homodimers formed, thereby reducing signaling via VEGFR2. On the other hand, VEGF-A/PlGF heterodimers can still bind VEGFR1, competing with VEGF-A and increasing the amount of unbound VEGF-A available for VEGFR2 binding (Autiero et al., 2003; Tarallo et al., 2010; Yang et al., 2013). It is therefore extremely difficult to predict the effects of PlGF because it can either increase VEGFR2 signaling (by freeing up VEGF-A from VEGFR1) or decrease VEGFR2 signaling (by trapping VEGF-A in VEGF-A/PLGF heterodimers). What mechanism dominates under which circumstances is currently not known, but the complexity of the system may be responsible for some of the apparent contradictory in vivo findings about PlGF described below in section 3.

To complicate matters further, VEGF-A/PlGF heterodimers, like VEGF-A, can bind VEGFR1/2 heterodimers (Autiero et al., 2003). In fact, VEGFR1/2 heterodimers exist even in the absence of VEGF-A/PlGF heterodimer ligands (Fig. 2) (Autiero et al., 2003). Computational modeling has shown that, in cells expressing both receptors, VEGFR1/2 heterodimers comprise 10–50% of active, signaling VEGFR complexes, and form preferentially over VEGFR1 homodimers when VEGFR2 is more abundant (Mac Gabhann and Popel, 2007). It has been suggested that VEGFR1/2 heterodimers reduce signaling via VEGFR2 homodimers (Cai et al., 2017; Cudmore et al., 2012), but the signal transduction properties of VEGFR heterodimers are currently not well characterized and their functional roles in an in vivo context are, at this stage, very difficult to predict. Nevertheless, some of the known biological effects of VEGF-A/PlGF and VEGFR1/2 heterodimers are discussed further in sections 3.1.2 and 3.2.

#### 2.2.5. VEGF co-receptors

Neuropilins NRP1 and NRP2 were first identified as co-receptors for semaphorin and VEGF signaling during neural and vascular development (Giger et al., 1998; Gu et al., 2003; Sulpice et al., 2008). NRPs are transmembrane proteins with a small cytoplasmic domain that lack intrinsic catalytic function (Fujisawa et al., 1997). The larger heparin-binding members of the three VEGF ligands (VEGF-A, VEGF-B, and PlGF) are able to bind NRP1 and NRP2 (Makinen et al., 1999; Migdal et al., 1998), bridging VEGFRs and NRP1 or NRP2 to create holoreceptor complexes (Pellet-Many et al., 2008; Wild et al., 2012) and inducing intracellular trafficking of VEGFR2, which is a critical event for downstream signal transduction (Simons et al., 2016). In ECs, NRPs modulate VEGFR signaling, enhancing migration (Soker et al., 1998) and survival (Favier et al., 2006). In addition, NRP1 has also been implicated in the spatial organization of ECs within angiogenic sprouts (Fantin et al., 2013; Kawamura et al., 2008) and in mouse models of pathological choroidal and retinal neovascularization (Dejda et al., 2014, 2016; Fernandez-Robredo et al., 2017).

The biological relevance of NRP1 for VEGF signaling has yet to be fully elucidated because mice with a mutant version of NRP1 that cannot bind VEGF develop normally (Gelfand et al., 2014). Furthermore, NRPs have been shown to bind other growth factors, such as TGF-β (Glinka and Prud’homme, 2008), fibroblast growth factor, and others (Uniewicz and Fernig, 2008; West et al., 2005). In ECs, NRP1 plays an important role during angiogenic sprouting, modulating differential responsiveness to TGF-β superfamily signaling independently of VEGF-A (Aspalter et al., 2015). The relevance of interactions between NRPs and other growth factors in the context of vascular biology remains to be established.

More recently, biochemical studies have revealed that the glycocalyx component endomucin interacts with VEGFR2 (independent of the presence of VEGF-A) and that knock-down of endomucin in cultured human retinal ECs using small interfering RNA blocks the biologic action of VEGF-A by preventing VEGFR2 internalization (LeBlanc et al., 2019; Park-Windhol et al., 2017). Preliminary studies indicate that VEGFR1 internalization also requires the presence of endomucin, but it is unclear if this applies to VEGFR1 homodimers or requires dimerization between VEGFR1 and VEGFR2.

### 2.3. Downstream signaling of VEGFRs

Upon ligand binding, conformational changes in the VEGFR intracellular domains lead to autophosphorylation of specific tyrosine residues. This allows binding of several signaling mediators such as phospholipase C gamma, non-receptor TKs such as Src, and adaptor proteins, such as those containing the Src homology 2 domain. Consequently, VEGFR1 and VEGFR2 activation induce signaling pathways that are normally activated by TK receptors (Fig. 1b), such as extracellular signal-regulated kinase (ERK)/mitogen-activated protein kinase (MAPK), phosphoinositide 3-kinase (PI3K)/Akt, and p38 (Jeltsch et al., 2013; Koch and Claesson-Welsh, 2012). Downstream signals from these receptors converge to cooperatively regulate transcription of different genes, leading to cell proliferation, migration, and survival, as well as controlling cell–cell contacts, cell–matrix adhesions, and cytoskeletal rearrangements (Fig. 1c), depending on cell type and biological context (Jeltsch et al., 2013; Koch and Claesson-Welsh, 2012).

To date, there is little comparative evidence regarding the relative activation of the different VEGFR1 signaling pathways following binding by VEGF-A and PlGF homodimers and heterodimers. However, there are important differences between the downstream signaling mechanisms induced by activation of VEGFR1 and VEGFR2. VEGFR2 has strong TK activity that induces a plethora of signals depending on which tyrosine becomes phosphorylated after ligand binding, whereas VEGFR1 has comparatively weak kinase activity (Koch and Claesson-Welsh, 2012; Meyer et al., 2006; Olsson et al., 2006), although some studies suggest potentially stronger downstream activity when PlGF binds VEGFR1 compared to VEGF-A (Roskoski, 2008). Like many other TK receptors, VEGFRs are internalized by clathrin-mediated endocytosis upon ligand binding and are subsequently proteolytically degraded (Pitulescu and Adams, 2014).

The downstream signaling of VEGFRs can be modified by VEGFR interactions. Specifically, PlGF is known to regulate intermolecular and intramolecular crosstalk between VEGFR1 and VEGFR2. For example, VEGFR1 activated by PlGF can trans-phosphorylate VEGFR2 (Autiero et al., 2003). Furthermore, signaling via VEGFR1/2 heterodimers can lead to outcomes that are different to VEGFR2 homodimer signaling (Cudmore et al., 2012). Activation of VEGFR1 by VEGF-A and PlGF homodimers, as well as by VEGF-A/PlGF heterodimers, induces overlapping pathways, but also a distinct downstream response. The pattern of VEGFR1 tyrosine phosphorylation differs in a ligand-dependent manner. PlGF, but not VEGF-A, directly stimulates ECs through the phosphorylation of tyrosine residues 1213 (Autiero et al., 2003) and 1309 (Dewerchin and Carmeliet, 2012; Fischer et al., 2008; Koch and Claesson-Welsh, 2012; Selvaraj et al., 2003). VEGFR1 phosphorylation is stimulated by VEGF-A but fails to alter the gene expression profile of mouse capillary ECs, whereas PlGF stimulation induces the expression of more than 50 genes (Roskoski, 2008). Furthermore, VEGF-A/PlGF and VEGF-A, but not PlGF, induce Akt-mediated cyclin-dependent kinase inhibitor 1B (p27Kip1) phosphorylation at residue Thr198 that is associated with its cytoplasmic retention and stimulation of cell motility (Apicella et al., 2018). Use of a synthetic heterodimer that does not occur naturally in humans, comprising VEGF-E (a non-mammalian protein) and PlGF, which specifically bind VEGFR2 and VEGFR1, respectively, activates the VEGFR1/2 heterodimer and highlights the ability of a receptor heterodimer to regulate EC homeostasis, migration, and vasorelaxation via the nitric oxide pathway (Cudmore et al., 2012).

It is clear from the research to date that the relationship between PlGF and VEGF-A and their interactions with the VEGFR1 and VEGFR2 receptors is remarkably complex and the resulting downstream effects are multifaceted. What is much less clear are the factors that determine which downstream signaling pathways are activated/modulated, and how these translate into functional responses, depending on the circumstances. However, the recent emergence of single cell analysis is likely to facilitate progress in this field in the near future.

## 3. Biological functions of VEGFR1

Activation of the signaling cascades downstream of VEGFRs can lead to numerous biological outcomes, depending on cell type and the expression profiles of VEGFRs/ligands in the tissue, as well as the presence of other growth factors and cytokines. Therefore, to understand the biological function of VEGFR1, it is important to consider all the signal-modulating mechanisms outlined in section 2.

### 3.1. Angiogenesis

VEGF-A is the prototypical VEGF family member and stimulates angiogenesis via VEGFR2 activation in ECs in both physiological and pathological settings (Cebe-Suarez et al., 2006; Ferrara, 2009; Jakeman et al., 1992; Koch and Claesson-Welsh, 2012; Nagy et al., 2007). Indeed, homozygous VEGFR2 KO mice die at embryonic day 8.5 due to defective cardiovascular development (Shalaby et al., 1995). Importantly, even single allelic VEGF-A deficiency results in embryonic lethality (Carmeliet et al., 1996; Ferrara et al., 1996), indicating that VEGF-A–mediated vascular formation is strictly dose-dependent. During retinal development, oxygen demand in differentiating neurons induces VEGF-A expression in astrocytes and Müller glia, which stimulates sprouting and lumenization of new blood vessels, leading to the formation of superficial and deep layers of the retinal vasculature (Blanco and Gerhardt, 2013; Claxton and Fruttiger, 2003; Liu et al., 2006; Pierce et al., 1995; Selvam et al., 2018; Stone et al., 1995; Zhang et al., 2007). Likewise, VEGF-A signaling is also fundamental for retinal, subretinal, and choroidal neovascularization in various eye diseases (Campochiaro, 2015).

#### 3.1.1. VEGFR1 decoy activity during angiogenic sprouting

Endothelial cells in angiogenic sprouts display distinct phenotypes depending on their position. At the front of the sprout, tip cells “sense” their environment using long filopodia, essentially navigating along a VEGF-A gradient (Gerhardt and Betsholtz, 2005; Gerhardt et al., 2003; Jeltsch et al., 2013). VEGF-A increases delta-like 4 expression in tip cells, which in turn induces a stalk cell phenotype in more proximal ECs via Notch-mediated lateral inhibition (Blanco and Gerhardt, 2013; Hellstrom et al., 2007; Jakobsson et al., 2009), triggering down-regulation of VEGFR2 and upregulation of VEGFR1 in stalk cells. Accordingly, sequestration of VEGF-A by VEGFR1 on these stalk cells results in spatial restriction and fine tuning of VEGF-A signaling at the growing vascular front.

Pericytes associated with angiogenic sprouts also express transmembrane and soluble VEGFR1, and the genetic ablation or the biochemical inhibition of PlGF or VEGFR1 in tumor models have implicated a direct role of VEGFR1 signaling in pericyte recruitment and vessel stabilization (Cicatiello et al., 2015; Tarallo et al., 2010). On the other hand, the developing retinal vasculature of the pericyte-specific VEGFR1 KO mice displayed normal numbers of pericytes but increased numbers of ECs and angiogenic sprouts with abnormally expanded morphology, suggesting that VEGFR1 on pericytes spatially restricts VEGF signaling at the angiogenic sprout (Eilken et al., 2017). Thus, the main role of VEGFR1 on pericytes awaits further investigation.

#### 3.1.2. Activity of VEGFR1 ligands in angiogenic sprouting

The role of direct VEGFR1 mediated signaling is not obvious, because (1) *VEGFR1-TK*^−/−^ mice survive and do not develop any obviously detrimental phenotypes (Hiratsuka et al., 1998); (2) *Pgf* KO mice display only very subtle developmental angiogenic abnormalities, with small and transient reductions in angiogenic sprouting during retinal and brain vascularization (Kay et al., 2017; Luna et al., 2016), and (3) VEGF-B KO mice also appear to have a largely normal phenotype (Bellomo et al., 2000). Nevertheless, the most pronounced effects of disrupting the VEGFR1 signaling axis have been observed in the context of pathologies. Tumor growth (Apicella et al., 2018; Carmeliet et al., 2001), arthritis (Yoo et al., 2009), and recovery from heart (Luttun et al., 2002; Pipp et al., 2003) and limb ischemia (Gigante et al., 2006) were all reduced in the absence of PlGF-mediated signaling. Furthermore, angiogenesis in ischemic retinas and laser-injured choroids, as well as diabetes-induced retinal cell death, capillary degeneration, pericyte loss, and BRB breakdown were alleviated in *Pgf* KO mice or by pharmacologically inhibiting PlGF activity (Apicella et al., 2018; Carmeliet et al., 2001; Crespo-Garcia et al., 2017; Huang et al., 2015; Rakic et al., 2003).

In general, artificially increasing levels of VEGFR1 ligands can result in more obvious phenotypes, usually characterized by increased angiogenesis. For instance, transgenic mice overexpressing PlGF in the skin under the keratin-14 promoter have a substantial increase in the number and size of dermal blood vessels (Odorisio et al., 2002). Similarly, adenovirus-mediated *Pgf* transfer in ischemic heart and limb tissue elicits a strong angiogenic response that is comparable to that of VEGF-A (Luttun et al., 2002). Transcranial injection of adeno-associated virus vectors encoding *Pgf* induces a robust stimulation of angiogenesis and arteriogenesis in the central nervous system (Gaal et al., 2013).

However, findings have not been entirely consistent. For example, one study has indicated that reduction of VEGF-B activity (using VEGF-B KO mice or an anti-VEGF-B antibody) may improve diabetic readouts in mice (Hagberg et al., 2012), whereas another report suggested that diabetic disease hallmarks can be improved by increasing VEGF-B, rather than blocking its activity (Robciuc et al., 2016). Transgenic overexpression of PlGF in T cells under the CD2 promoter produced a significant reduction in placental angiogenesis that was linked to the inhibition of BRAF and activation of ERK (Kang et al., 2014), indicating that the effects of PlGF on angiogenesis are context-dependent. Moreover, reduced angiogenesis has been described after blocking PlGF in mouse tumor models (Van de Veire et al., 2010), but this was not confirmed by others (Bais et al., 2010).

Such conflicting results could arise from the complexity and context-dependency of VEGFR1 signaling and, as detailed in section 2, there are several molecular mechanisms that can lead to different outcomes. For example, increased PlGF production can increase VEGF-A activity and angiogenesis via competition for VEGFR1 binding, whereas, if PlGF is expressed in the same cell, formation of PlGF/VEGF-A heterodimers could result in reduced VEGFR2 homodimer activation. In fact, co-expression of VEGF-A and PlGF occurs in many cell types, including ECs and pericytes (Yonekura et al., 1999), fibroblasts (Green et al., 2001), macrophages (Bottomley et al., 2000), keratinocytes (Failla et al., 2000), and RPE cells (Klettner et al., 2015), making it difficult to delineate which biological activity is attributable to VEGF-A or PlGF homodimers versus PlGF/VEGF-A heterodimers (DiSalvo et al., 1995).

**Fig. 3.**
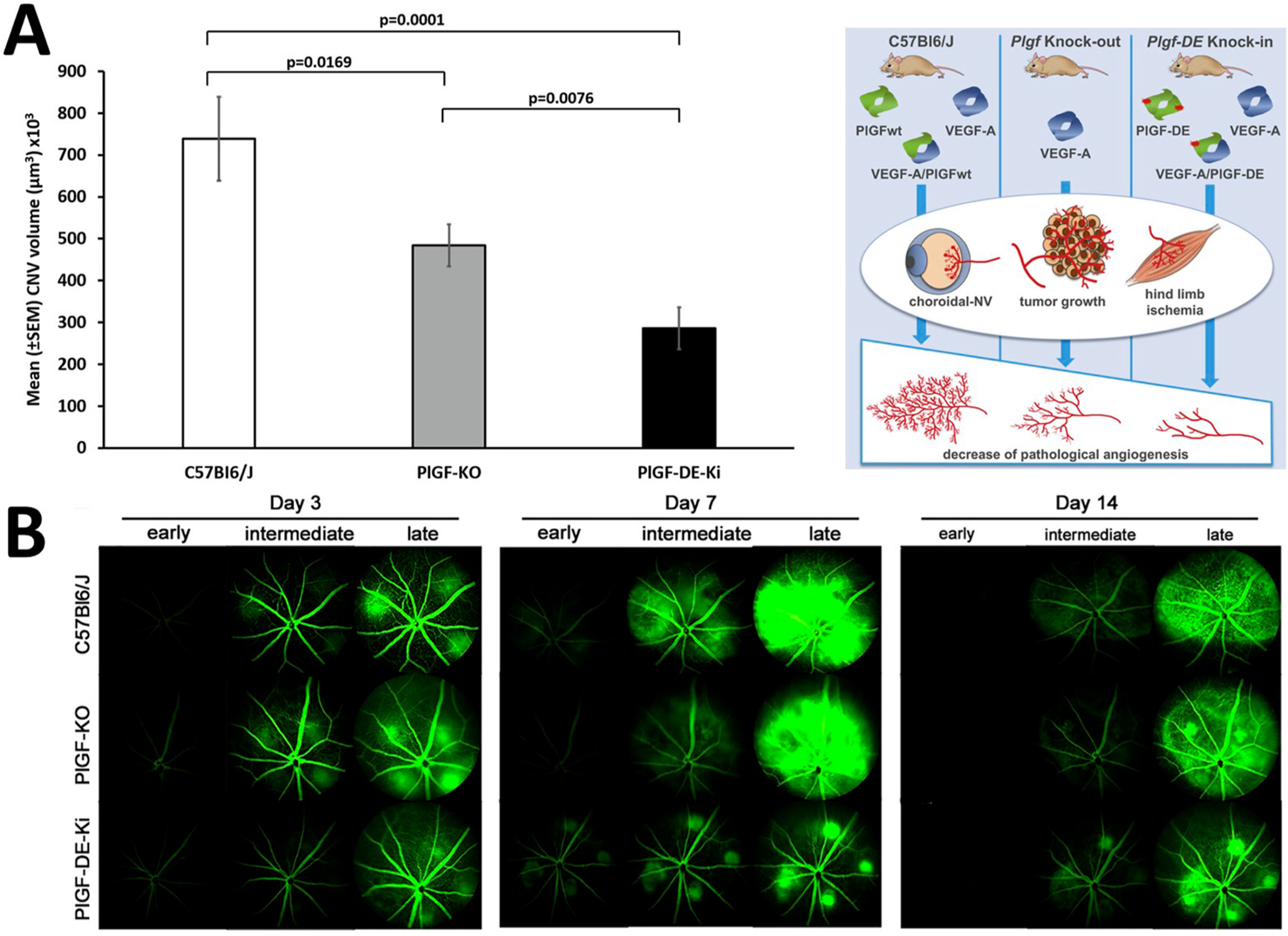
*Pgf*-DE-Ki mice, a fully functional *Pgf*-KO model achieved by knocking in the *Pgf*-DE variant unable to bind and activate VEGFR1, show robust reduction of CNV and protection from vascular leakage. **A.** CNV volumes measured 7 days after laser-induced damage by Isolectin B4 staining of RPE-choroid flat mounts. **B.** Q ualitative fundus fluorescein angiography in C57BL6/J, *Pgf*-KO, and *Pgf*-DE-Knock-in mice acquired at three different times (early – 1 min, intermediate – 5 min, late – 15 min) after intraperitoneal delivery of fluorescein at Days 3, 7, and 14 after laser-induced damage. Reproduced under Creative Common CC-BY license (Apicella et al., 2018).

Recent work by Apicella et al. suggests that PlGF/VEGF-A heterodimers do have a positive effect on angiogenesis (Apicella et al., 2018), which may be mediated by a VEGFR1/2 heterodimer (Yang et al., 2013). The *Pgf*-DE knock-in mouse, generated by knocking into the *Pgf* locus a variant (*Pgf*-DE) that is unable to bind and activate VEGFR1 (Tarallo et al., 2010), allowed the investigation of the effects of the complete loss of function of PlGF, as these mice produce homodimers of PlGF-DE and heterodimers of VEGF-A/PlGF-DE that are inactive (Apicella et al., 2018). These mice showed significant impairment of angiogenesis in tumor growth, hind limb ischemia, and CNV compared with *Pgf* KO and wild-type mice (Fig. 3). Moreover, in a laser-induced CNV model, these mice show a large reduction in vascular leakage. In parallel, the recombinant VEGF-A/PlGF heterodimer is able to rescue vascularization and vascular leakage to an extent that is similar to that of recombinant VEGF-A (Apicella et al., 2018). These results highlight the central role of the VEGF-A/PlGF heterodimer on vascular leakage and neo-angiogenesis stimulation during CNV.

### 3.2. Vascular permeability

Optimal functioning of the neuronal cells of the retina necessitates a tightly regulated environment in each of the functional compartments. In the healthy state, this is achieved through an intact BRB that provides such an environment and, through the cellular barriers, allows the uptake of essential nutrients and elimination of discarded metabolites. As stated in the introduction, there are two distinct barriers protecting the retina. The outer BRB, consisting of the RPE, regulates transport between the choriocapillaris and the outer retina (Fields et al., 2020). The inner BRB regulates transport across the retinal capillaries within the inner retina and is composed of a single layer of tightly adherent endothelial cells, a basal lamina, and surrounding pericytes, astrocytes, and microglia (Díaz-Coránguez et al., 2017; Klaassen et al., 2013). In retinal capillaries, pericytes contribute to maintenance of barrier function, not only by providing mechanical support, but also by communicating with ECs via paracrine signals and direct cell–cell contact (Armulik et al., 2011).

Vascular permeability is thought to be mediated via two mechanisms: the transcellular route and the paracellular route. The transcellular route involves vesicular transport and the formation of channels from vesicles or vacuoles, the vesiculo-vacuolar organelle. The paracellular route is based on transient changes in junctions between ECs (adherens and tight junctions) (Wettschureck et al., 2019). VEGF-A was initially identified as a factor secreted by tumor cells that induces vascular permeability (Senger et al., 1983). The process is mediated via VEGFR2 and is likely to involve the tyrosine phosphorylation, internalization, and degradation of vascular endothelial-cadherin, a major component of endothelial adherens junctions (Dejana, 2004; Dejana and Orsenigo, 2013; Dejana et al., 2008). It may also be attributable to transcellular extravasation via EC vesicles (Lin et al., 2007). VEGF-A/VEGFR2-mediated modulation of vascular permeability and plasma extravasation also involves the activation of endothelial nitric oxide synthase (eNOS) and production of nitric oxide (Papapetropoulos et al., 1997; Ziche et al., 1997b).

Despite the fact that vascular permeability is generally thought to be mediated primarily by VEGF-A/VEGFR2, there is also evidence for a role for VEGFR1, PlGF, and VEGF-A/PlGF heterodimers. VEGFR1 activation controls vascular permeability via eNOS activation, as is the case for VEGFR2. In addition, there is a functional link between PlGF and eNOS activation. The phosphorylation of eNOS, which occurs downstream of ERK and Akt activation (Feliers et al., 2005; Hisamoto et al., 2001), has been directly associated with the activation of VEGFR1 (Bussolati et al., 2001), and is supported by findings that vascular leakage in mouse models can be reduced by blocking PlGF (Carmeliet et al., 2001) or VEGFR1 (Huang et al., 2011). An in vitro study of the mechanism of high-glucose-induced damage to retinal ECs concluded that PlGF was signaling through the Erk1/2-NOS axis via VEGFR1 (Jiao et al., 2019). A recent study has reported that PlGF directly disrupts barrier function by suppression of glucose-6-phosphate dehydrogenase and peroxiredoxin, acting through glutathione peroxidase and phospholipase A_2_ activity (Huang et al., 2019a). VEGF-A also induces vasodilation, mainly through VEGFR1 signaling, with the involvement of VEGFR1/2 receptor heterodimerization (Cudmore et al., 2012), a finding that has been corroborated using the *Pgf*-DE knock-in mouse model (Apicella et al., 2018) described in section 3.1.2.

In ECs, VEGFR1 and VEGFR2 are distributed luminally and abluminally, respectively, suggesting that the highly polarized signaling depends on the receptor position (Hudson et al., 2014). The majority of VEGFR1 is localized to the apical or luminal sides of retinal microvascular ECs, whereas VEGFR2 is predominantly located on the basal or abluminal sides (Hudson et al., 2014); however, this generalization has been the subject of recent debate (Blaauwgeers et al., 1999; Dragoni and Turowski, 2018; Van Bergen et al., 2019). Thus, luminal and abluminal endothelial surfaces display differential functionality, with luminal VEGFR1 activation via circulating VEGF-A leading to Akt activation and facilitation of EC survival and abluminal VEGFR2 activation via tissue-borne VEGF-A leading to increased permeability via p38 (Hudson et al., 2014). This suggests important roles for the MAPK and PI3K/Akt pathways downstream of VEGFR2 in angiogenesis and permeability. The impact of differential apicobasal signaling, as a consequence of VEGFR1 or VEGFR2 activation, should be considered when working with experimental models of retinal disease (Cao et al., 2010; Liu et al., 2017).

### 3.3. VEGFR1 and inflammation

Although not necessarily the primary etiopathogenic factor, persistent microinflammation can cause considerable collateral damage in many age-related chronic diseases, fueling further inflammation (Nathan and Ding, 2010) and is a known contributor to retinal pathology as well (Rashid et al., 2019). In the affected tissues, inflammation is often associated with the persistence of mononuclear phagocytes, a family of cells that includes circulating monocytes, tissue-resident macrophages, and monocyte-derived inflammatory macrophages (Nathan and Ding, 2010). VEGFR1 and PlGF are known to be involved in the inflammatory pathways (Shibuya, 2015). Genetic ablation of the VEGFR1 TK domain in mice allows normal vascular development but significantly suppresses VEGF-induced macrophage migration (Hiratsuka et al., 1998). There is strong evidence that PlGF and VEGFR1 signaling can influence how immune cells affect tumor growth and metastasis (Albonici et al., 2019; Ceci et al., 2020; Incio et al., 2016; Kim et al., 2012; Muramatsu et al., 2010; Qian et al., 2015) as well as cardiovascular disorders (Luttun et al., 2002; Raisky et al., 2007; Roncal et al., 2010) or rheumatoid arthritis (Murakami et al., 2006).

PlGF/VEGF-A heterodimers have been detected in synovial fluid samples from patients with inflammatory arthropathy and in human keratinocytes during wound healing, with levels of PlGF and VEGF-A in synovial fluid correlating significantly with total leukocyte and neutrophil counts (Bottomley et al., 2000). An increase in inflammatory cytokine production after VEGFR1 activation in mononuclear phagocytes has also been observed in patients with rheumatoid arthritis, in which fibroblast-like synoviocytes produce high levels of PlGF (Yoo et al., 2009). PlGF-induced VEGFR1 activation increased TNF-α and IL-6 expression, whereas TNF-α and IL-1β upregulated VEGFR1 (Yoo et al., 2009).

In the retina, circulating monocytes, macrophages, and so-called resident microglia — which are not true glial cells but specialized, resident mononuclear phagocytes — are constantly engaged in the surveillance of their surrounding tissue (Akhtar-Schafer et al., 2018; McMenamin et al., 2019). Retinal microglia are maintained mostly by self-renewal through the entire life span but can also be replenished from extraretinal sources (Huang et al., 2018). This contrasts with continuous replenishment of choroidal macrophages by circulating monocytes (O’Koren et al., 2019). During development, microglia contribute to the refinement of the neural circuits (Akhtar-Schafer et al., 2018; McMenamin et al., 2019; Reyes et al., 2017) and also influence morphogenetic patterning of the vascular network, facilitating vascular anastomosis (Fantin et al., 2010; Kubota et al., 2009; Rymo et al., 2011). The VEGFR1 signal contributes but is not indispensable for the development of retinal microglia and the superficial retinal vascular networks (Ogura et al., 2017). In contrast, microglia in the deep retinal layer express VEGFR1, and the decoy function of this receptor neutralizes circulating PlGF/VEGF-A, thereby reducing angiogenic branching of the deep retinal vessels (Stefater et al., 2011).

In addition to microglia, which are the most common immune cells in the retina, the retina also contains ionized calcium-binding adapter molecule 1 (Iba1)-negative perivascular macrophages found on the abluminal aspect of the vascular endothelial basal lamina, that are closely associated with pericytes and Müller cells in the deeper retina (Mendes-Jorge et al., 2009). The microglia and perivascular macrophages can also be differentiated from one another since the macrophages express BM8 and MOMA-2 antigen epitopes, which are not expressed by microglia (Mendes-Jorge et al., 2009) and their position relative to retinal blood vessels indicate their involvement in the preservation of the BRB as well as the immune defense against blood-borne pathogens (McMenamin et al., 2019). On breakdown of the BRB and photoreceptor degeneration, the macrophages migrate to the site of damage as shown in various models of retinopathy (Roubeix et al., 2019; Saban, 2018; Sennlaub et al., 2013). Retinal injury can activate microglia and trigger the secretion of inflammatory mediators, such as CC chemokine ligand 2 (CCL2, also known as monocyte chemoattractant protein-1), IL-1β, IL-6, and TNF-α (Grossniklaus et al., 2002; Oh et al., 1999), which can further aggravate retinal injury (Langmann, 2007).

In the disease state, activation of VEGFR1 results in the production by macrophages and microglia of proinflammatory and proangiogenic mediators in the retina (Carmeliet et al., 2001; Crespo-Garcia et al., 2017; Fischer et al., 2008; Rakic et al., 2003; Ziche et al., 1997a). Furthermore, PlGF may stimulate VEGFR1-dependent migratory pathways of monocytes more efficiently than does VEGF-A (Cicatiello et al., 2015; Clauss et al., 1996). Both activated microglia and monocyte-derived macrophages are assumed to upregulate VEGFR1 in various sites including the retina (Barleon et al., 1996; Ogura et al., 2017). VEGFR1 activation in these mononuclear phagocytes upregulates their production of pro-inflammatory and pro-angiogenic cytokines, such as CCL2, IL-1β, IL-6, TNF-α, and VEGF-A (Murakami et al., 2006; Selvaraj et al., 2003).

However, there remains a large gap in our understanding of how exactly VEGFR1 signaling in inflammatory cells contributes to retinal vascular pathology. As outlined in this section, various types of inflammatory cells of different origins (i.e. resident versus invading) and in different states of activation are found in the retina. Thus, whilst certain features of the pathologies provide hints of inflammatory cell involvement, further study of the many levels of intricacy might resolve some of the apparent experimental paradoxes in the VEGFR1 literature.

## 4. Role of VEGFR1 in retinal vascular disease

The seminal discovery of increased VEGF levels in ocular fluids of patients with retinal eye diseases (Adamis et al., 1994) introduced an era of anti-VEGF therapy in diseases such as DR, retinal vascular occlusions, retinopathy of prematurity (ROP), neovascular AMD and others. Currently in use are, 1) aflibercept and conbercept* (fusion proteins consisting of the ligand binding portions of VEGFR1 and VEGFR2 extracellular domains fused to the Fc portion of human IgG), 2) bevacizumab* (a full-length anti-VEGF monoclonal antibody), 3) ranibizumab (an anti-VEGF monoclonal antibody Fab fragment), and 4) brolucizumab (a single-chain antibody fragment), recently approved for neovascular AMD (Markham, 2019) (see Table 1).

Aflibercept and conbercept* bind all known VEGFR1 ligands, VEGF-A, PlGF, and VEGF-B, unlike ranibizumab, bevacizumab,* and brolucizumab, which are VEGF-A specific (de Oliveira Dias et al., 2016; Papadopoulos et al., 2012). It is tempting to hypothesize that some observed differences between the clinical effects of anti-VEGF agents may result from differences in their targets, i.e. the binding of VEGF-A, VEGF-B and PlGF versus just VEGF-A, but given the complexity of VEGFR1 signaling and the impact of other factors like pharmacokinetics, binding affinities, and dosing strategies, this hypothesis remains open to further investigation. Furthermore, despite significant advances in our understanding of the molecular and cellular aspects of VEGF receptors and their ligands, it is only more recently that research has focused on elucidating the effects of VEGFR1 versus VEGFR2 in the pathophysiology of retinal diseases.

### 4.1. Diabetic retinopathy

Diabetic retinopathy is the leading vision-threatening disease in the working-age population globally (Yau et al., 2012). Over years of hyperglycemic episodes, the accumulation of insults, including advanced glycation end products and oxidative stress, damages retinal blood vessels and neural cells (Duh et al., 2017). About one-third of diabetic patients display non-proliferative DR (NPDR) characterized initially by intraretinal microvascular abnormalities and retinal microaneurysms, and additionally by retinal hemorrhage, edema, and exudative lipoprotein deposits (known as hard exudates) (Duh et al., 2017; Wong et al., 2018). In more severe cases, capillary non-perfusion and subsequent tissue ischemia can lead to retinal microinfarctions and collateral vessel formation and ultimately retinal neovascularization (Duh et al., 2017; Wong et al., 2018), thus evolving into PDR. PDR is distinguished by the growth of retinal neovascularization extending into the vitreous cavity, ultimately resulting in vision-impairing vitreous hemorrhage and tractional retinal detachment (Duh et al., 2017; Wong et al., 2018). A further complication is diabetic macular edema (DME), which affects central vision in any DR, with an estimated prevalence of approximately 7% among people with diabetes (Yau et al., 2012).

For DME treatment, intravitreally injected anti-VEGFs have been demonstrated to be effective in multiple clinical trials and have subsequently been widely adopted worldwide (Ferrara and Adamis, 2016; Sivaprasad et al., 2017; Wong et al., 2018; Writing Committee for the Diabetic Retinopathy Clinical Research et al., 2015). Anti-VEGF agents are effective in reducing the edema associated with the increased vascular permeability in the retina and in improving the vision of patients with DME (Heier et al., 2016; Nguyen et al., 2012). This clearly illustrates the pivotal role VEGF-A plays in angiogenesis as well as in vascular permeability. However, the exact mechanisms behind this remarkable success story are less well understood. Additionally, as outlined in the previous section, VEGF-A does not only play a role in endothelial cell behavior but is also relevant for inflammatory cells, whose contribution to DR is overshadowed by the current focus on vascular phenotypes. Furthermore, the roles of the different VEGF ligands within the context of human retinal pathology have not yet been properly elucidated. Nevertheless, there are clinical observations and animal experiments that allow us to hypothesize about potential mechanisms.

Initiation of the inflammatory response that is linked to the early stages of the pathogenesis of DR was demonstrated initially by leukocyte-mediated endothelial cell injury and death in animal models (Joussen et al., 2001, 2004). Indeed, one of the most compelling arguments for an involvement of inflammation in DR is the well-established potency of corticosteroids in the treatment of DME (Rittiphairoj et al., 2020; Whitcup et al., 2018; Wykoff, 2017). Interestingly, there is evidence that benefits of steroid treatment are not limited to just edema but may also slow down development of PDR and overall progression of DR (Pearson et al., 2011; Querques et al., 2017; Wykoff et al., 2017), suggesting that inflammatory mechanisms (such as persistent low-grade inflammation) make a causal contribution to DR (Kinuthia et al., 2020). This is consistent with a large collection of clinical studies showing increased ocular levels of inflammatory mediators, including IL-1β, IL-6, IL-8, TNF-α, and CCL2, in NPDR, DME and PDR (Bolinger and Antonetti, 2016; Chernykh et al., 2015; Funatsu et al., 2012; Kovacs et al., 2015; Mao and Yan, 2014; Mesquida et al., 2019; Rubsam et al., 2018; Tang and Kern, 2011; Wykoff, 2017; Zhou et al., 2012).

Considering the likely contributions from inflammatory cells in DR and the role of VEGF signaling in inflammatory cells (as covered in section 3), a key emerging question is whether the benefits of anti-VEGFs in ophthalmic practice are based only on the well-established effects on vessels or if they are also acting on inflammatory cells, and beyond that, whether signaling via VEGFR1 may be relevant. For the first part of this question there is considerable clinical evidence showing that anti-VEGFs can reduce inflammatory cytokines in diabetic eye disease. For instance, aflibercept injections not only suppressed levels of VEGF-A in DME patients but also reduced inflammatory cytokines such as IL-6, IL-1β and others (Mastropasqua et al., 2018). Similarly, a study on PDR patients receiving aflibercept showed reduced levels of IL-6, IL-8, IL-10 and IL-1β in the vitreous (Raczyńska et al., 2018). Likewise, ranibizumab treatment reduced levels of IL-1β, IL-8, IL-10, CCL2 and TNF-α in DME patients (Lim et al., 2018). However, it is not known yet whether the changed cytokine levels are a direct result of VEGF signaling inhibition or whether they are an indirect consequence of reduced vascular pathology.

The functional role of VEGFR1 in inflammation within the context of human diabetic eye disease remains to be fully understood. Looking at the VEGFR1 specific ligand, PlGF, is certainly a path to explore. In human eyes with DR, PlGF is elevated in addition to VEGF-A (Ando et al., 2014; Noma et al., 2015, 2017), and there are significant increases in the levels of both VEGF-A and PlGF in vitreous samples from eyes of patients with increasing levels of ischemia, i.e. from the normal to diabetic state, or from PDR to neovascular glaucoma (Kovacs et al., 2015; Patel, 1989). Whilst the mere presence of elevated PlGF levels in the vitreous does not prove a functional involvement in DR pathology, there appears to be an association between PlGF levels and progressive disease severity in DR and RVO (Noma et al., 2015). Mechanistic insights may be gained by comparing the clinical effects of drugs that target specifically VEGF-A (e.g. ranibizumab) versus the ones that in addition also target the VEGFR1 specific ligands PlGF and VEGF-B (e.g. aflibercept), although this is not straightforward either. Current clinical trials usually focus on visual acuity or retinal thickness under therapy and therefore an approach more focused on inflammation markers is required to shed more light on this topic.

An intriguing piece of clinical evidence comes, however, from a subtle feature in retinal OCT images in patients with DME that might be useful for the assessment of inflammation in vivo. In some patients with early DR, small well-demarcated, hyperreflective foci have been identified (Yu et al., 2019). Such deposits are located within walls of intraretinal microaneurysms and in some cases distributed throughout the retinal layers. Various etiologies have been suggested regarding the possible nature of these hyperreflective foci and it is suggested that they represent lipoproteins or lipid-laden macrophages, indicating extravasation and/or neuroinflammation, as an early subclinical sign of barrier breakdown in DME (Bolz et al., 2009). In early stages of DME with few or no funduscopically visible exudates, the number of hyperreflective spots, as visualized by OCT, decreased significantly after either anti-VEGF or steroid treatment and correlated with functional data. DME with high number of hyperreflective spots showed better morphologic and functional results (in terms of retinal sensitivity) if treated, at least initially, with steroids versus a selective VEGF-A inhibitor (ranibizumab) (Frizziero et al., 2016; Vujosevic et al., 2017). However, the hyperreflective spots cannot be used as a true proxy for inflammation until their cellular nature has been established more firmly.

The target specificity of currently used anti-VEGF drugs might also yield some hints about the pathobiological function of VEGFR1 in DR. There are numerous studies in DME patients showing significant differences between ranibizumab and aflibercept when looking at certain clinical readouts, with aflibercept showing higher efficacy or longer lasting treatment effects (Bhandari et al., 2020; Jampol et al., 2016; Kaldirim et al., 2019; Ozkaya et al., 2020; Sarda et al., 2020; Shimizu et al., 2017). Furthermore, comparisons between aflibercept and bevacizumab* had similar outcomes (American Academy of Opthalmology, 2019; Virgili et al., 2018; Wells et al., 2015; Wells et al., 2016). For example, some studies have found that aflibercept was statistically superior in vision gains compared with ranibizumab and bevacizumab* (Protocol T) (Wells et al., 2015, 2016). These outcome differences may be explained by several factors, such as anti-VEGF-A relative potency/binding affinity (Papadopoulos et al., 2012), specificity for VEGF-A only versus VEGFR1 blockade through binding of VEGF-A, PlGF and VEGF-B (Papadopoulos et al., 2012), duration of intraocular VEGF suppression (Fauser and Muether, 2016; Fauser et al., 2014; Muether et al., 2013), ocular pharmacokinetics (Do et al., 2020; Krohne et al., 2008, 2012) and drug formulation. The higher binding affinity, multiple molecular targets and ocular pharmacokinetics of aflibercept may be contributing factors to the observed differences in clinical outcomes. While the respective relative importance of these factors is unknown, these differences have been considered significant enough to define clinical practice guidelines (American Academy of Opthalmology, 2019; Cheung et al., 2018b; Schmidt-Erfurth et al., 2017). In this respect it will be interesting to see direct comparisons between aflibercept or conbercept* and some of the more recently approved anti-VEGFs. For example, brolucizumab has a higher molarity of VEGF-A binding sites compared with aflibercept but only binds VEGF-A and not PlGF and VEGF-B. Differences in future clinical trial outcomes might inform us further about the molecular effects of these drugs.

Despite the current absence of such comparative data it is intriguing that aflibercept has been shown to reduce overall DR progression in DME patients (Mitchell et al., 2018) and to improve DR severity in PDR patients (Nittala et al., 2020). This is remarkable because anti-VEGFs are generally seen to treat complications of DR (i.e. high VEGF levels) and not the underlying disease. It is possible that effects on inflammatory mechanisms could reduce overall progression and severity in DR. Future clinical studies measuring cytokines in aqueous humor from DR patients treated with aflibercept or conbercept* versus ranibizumab or brolucizumab are likely to add further insight here.

In addition to clinical observations, studies in animal models can inform us about potential functional roles of VEGFR1 and PlGF in retinal pathology. For instance, overexpression of PlGF in ciliary muscle of rats led to microaneurysms and vascular sprouts in the retinal vasculature, demonstrating the pathogenic potential of elevated PlGF levels (Kowalczuk et al., 2011). Similarly, PlGF injection into the rat eye vitreous caused sub-retinal fluid accumulation by opening RPE tight junctions (Miyamoto et al., 2007). Additionally, deletion of the *Pgf* gene in a type 1 diabetic mouse model (*Ins2*Akita mouse carrying a spontaneous point mutation in the *insulin 2* gene) (McLenachan et al., 2013) led to protection from capillary dropout, pericyte loss, and BRB breakdown (Huang et al., 2015). Furthermore, the absence of PlGF increased Akt phosphorylation and inhibited the HIF-1α–VEGF pathway, preventing retinal cell death, capillary degeneration, pericyte loss, and BRB breakdown, which highlights the critical role of PlGF and VEGFR1 in the development of DR. Increased expression of the tight junction molecule, ZO-1 and vascular endothelial-cadherin alongside sonic hedgehog and angiopoietin-1 also indicated additional protection associated with *Pgf* deletion (Huang et al., 2015). In contrast, expression of intracellular adhesion molecule (ICAM)-1, vascular cell adhesion molecule-1, CD11b, CD18 and retinal leukostasis were not inhibited in this study (Huang et al., 2015).

An alternative approach, using VEGFR1 neutralizing antibodies in mice with streptozotocin-induced diabetes, led to a reduction of leukostasis and various cytokines (including IL-1β), besides lowered vascular permeability (He et al., 2015). This is consistent with another study in streptozotocin-treated rats, demonstrating reduced TNF-α after intravitreally injected aflibercept (Lazzara et al., 2019). Moreover, it has also been shown in type 1 diabetes mouse models that leukocyte and macrophage infiltration was decreased by an anti-PlGF specific antibody or aflibercept, but not by VEGF-A specific antibodies, suggesting a specific role of PlGF in retinal inflammatory mechanisms (Van Bergen et al., 2017). One should keep in mind though, that models based on diabetic animals (typically rodents) have limitations. Although they mimic some aspects of human NPDR, other features (such as PDR) are not present. Furthermore, while these models can indicate what might be going wrong in human eyes, they cannot be used to validate human disease mechanisms.

**Fig. 4.**
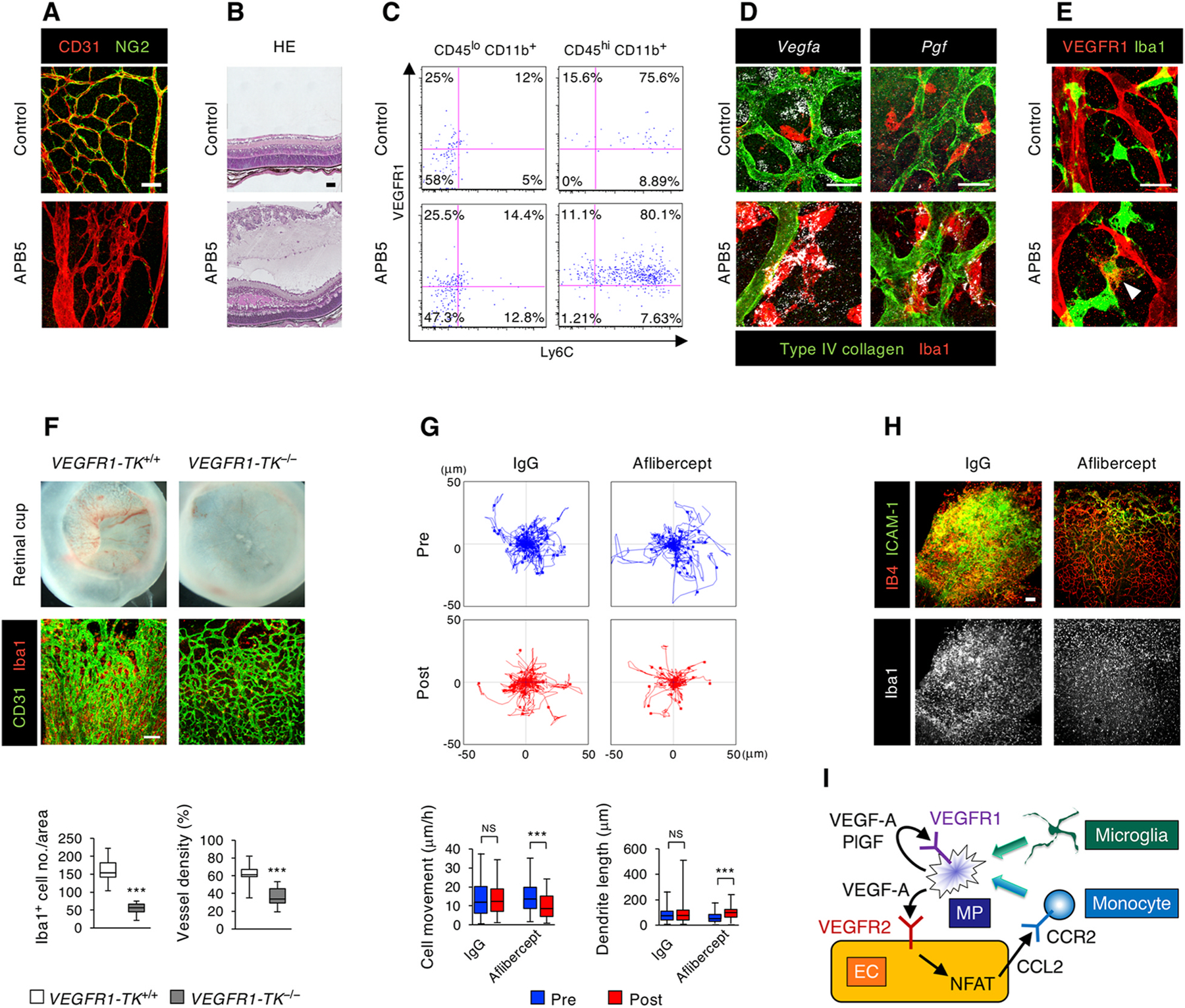
VEGFR1 signal in a mouse model of pericyte-deficient retinopathy (pups intraperitoneally injected with an anti-platelet-derived growth factor receptor β monoclonal antibody [clone APB5 in **A–H**] or control phosphate-buffered saline **[A–E]**) at postnatal day [P]1). **A.** Labeling of retinal endothelial cells (ECs) and pericytes (PCs) at P5 by whole-mount immunohistochemistry (WIHC) for CD31 and NG2, respectively. Note the absence of PCs and disorganized vascular networks in the APB5-treated retina. **B.** Hematoxylin and eosin (HE) staining of paraffin sections from P10 retinas showing edema and hemorrhage in the APB5-treated retina. **C.** Flow cytometry in P8 retinas. Tissue-resident microglia and inflammatory mononuclear phagocytes (MPs) are represented by CD45^lo^loCD11b^+^ and CD45^hi^CD11b^+^ cells, respectively. Note the high VEGFR1 expression level in CD45^hi^CD11b^+^Ly6C^+^ MPs from the APB5-treated retinas. **D.** Retinal whole-mount in situ hybridization for *Vegfa* (left) and *Pgf* (right) at P8 in conjunction with labeling of vascular basement membranes and MPs by WIHC for type IV collagen and Iba1, respectively. Note the upregulation of *Vegfa* and *Pgf* in perivascular MPs of the APB5-treated retinas. **E.** VEGFR1 reporter expression in P8 retinas from *Vegfr1-BAC-DsRed* mice in conjunction with WIHC for Iba1. Note the VEGFR1-expressing MP (arrowhead) in the APB5-treated retina. **F.** Retinal cups (upper) and WIHC for CD31 and Iba1 (lower) at P11 in APB5-treated *VEGFR1-TK* mice. Note the suppression of retinal edema and MP infiltration even without PC coverage in *VEGFR1-TK*^−*/*−^mice. The graphs show the number of Iba1^+^ cells per area and the vessel density (*n* = 20). **G.** The trajectory of MPs in APB5-treated retinas from P8 *Cx3cr1-GFP* mice. After 3 h ex vivo imaging, retinas were treated with control IgG or aflibercept, and further monitored for 3 h. The graphs show quantification of cell body movement velocity (Pre IgG, *n* = 68; Post IgG, *n* = 56; Pre VEGF Trap, *n* = 52; Post VEGF Trap, *n* = 47) and total dendrite length per cell (Pre IgG, *n* = 40; Post IgG, *n* = 34; Pre VEGF Trap, *n* = 43; Post VEGF Trap, *n* = 33). **H.** Labeling for isolectin B4 (IB4), ICAM-1, and Iba1 at P13 in APB5-treated retinas after intravitreal injections of control IgG or aflibercept at P7. Note the normalization of vascular networks with reduced MP infiltration after aflibercept injection. **I.** Schematic diagram of EC-MP interactions in PC-deficient retina. In ECs, activation of nuclear factor of activated T cells (NFAT) leads to upregulation of CCL2, which subsequently facilitates the influx of circulating CCR2^+^ monocytes. The infiltrating monocytes and activated microglia contribute to generation of inflammatory MPs, which secrete VEGF-A and PlGF, and activate VEGFR1 in MPs and VEGFR2 in ECs. The VEGF-A–VEGFR2 signal further activates NFAT. This positive feedback loop sustains breakdown of the blood-retina barrier. In box-and-whisker plots, median (line within the box), upper and lower quartile (bounds of the box), with minimum and maximum values (bars) are shown. ***p < 0.001; NS, not significant, by Student’s *t*-test. Scale bars, 50 μm (A and B); 20 μm (D and E); 100 μm (F and H). Adapted from Ogura et al. (2017) with permission from American Society for Clinical Investigation.

In histological analyses of human diabetic eyes, the loss of pericytes in the retinal vasculature is one of the first cellular pathologies that has been recognized (Cogan et al., 1961), and it is assumed that pericyte dropout is a key driver of vascular abnormalities in DR (Arboleda-Velasquez et al., 2015). In line with this notion, pericyte loss can be seen in some animal models of diabetes (Robinson et al., 2012). The consequences of pericyte loss in the retinal vasculature can also be studied in diabetes-independent models. For example, pericyte recruitment to growing retinal vessels can be efficiently disrupted by genetic or pharmacologic tools leading to disorganized vascular patterning with microaneurysms, edema, and hemorrhage (Enge et al., 2002; Kitahara et al., 2018; Klinghoffer et al., 2001; Kusuhara et al., 2018; Lindblom et al., 2003; Park et al., 2017; Uemura et al., 2002; Valdez et al., 2014) as shown in Fig. 4A and B.

Interestingly, in addition to the vascular pathologies, pericyte deficiency can also lead to inflammatory phenotypes via the activation of nuclear factor of activated T (NFAT) cells in ECs, which upregulates a series of inflammatory mediators and leukocyte adhesion molecules including CCL2 and ICAM-1, resulting in influx of CCR2-expressing monocytes and perivascular infiltration of CD45^hi^CD11b^+^Ly6C^+^ mononuclear phagocytes (Fig. 4C) (Ogura et al., 2017). These mononuclear phagocytes, which might also comprise activated microglia, displayed amoeboid cell bodies with fewer dendrites that physically contacted the denuded ECs (Ogura et al., 2017). In contrast to the tissue-resident microglia, mononuclear phagocytes infiltrating into pericyte-deficient retinas exhibited elevated VEGF-A, PlGF, and VEGFR1 (Fig. 4, C–E), which is indicative of VEGFR1 activation in an autocrine or paracrine manner (Ogura et al., 2017). ECs devoid of pericytes revealed increased VEGFR2, which would be expected to be activated by VEGF-A derived from mononuclear phagocytes and exacerbate vascular hyperpermeability (Ogura et al., 2017). In *VEGFR1-TK*^−*/*−^ mice, retinal edema and mononuclear phagocyte infiltration were reduced even after pericyte depletion (Fig. 4F) (Ogura et al., 2017). In addition, time-lapse imaging of ex vivo explants of pericyte-deficient retinas demonstrated that aflibercept reduced the motility of mononuclear phagocytes and recovered their dendrite formation (Fig. 4G) (Ogura et al., 2017). Furthermore, intravitreally injected aflibercept suppressed mononuclear phagocyte infiltration and vascular leakage in the pericyte-deficient retina (Fig. 4H). Together, as shown in Fig. 4I, these observations indicate a positive feedback loop between ECs and mononuclear phagocytes in pericyte-deficient retina, in which VEGFR1 signaling facilitates cell motility of mononuclear phagocytes. Thus, despite the underlying complexity of multiple signaling pathways mediating interactions between at least three different cell types, we can conclude that in this setting, simultaneous neutralization of VEGF-A and PlGF can effectively block the cycle of BRB breakdown.

### 4.2. Retinal vascular occlusions

Retinal ischemia due to vascular occlusion occurs most commonly in branch retinal vein occlusion (BRVO) and central retinal vein occlusion (CRVO) but may also appear as a complication in hemoglobinopathies (sickle cell disease and thalassemia), peripheral ischemic retinopathies such as Eales’ disease, familial exudative vitreoretinopathy, sickle cell retinopathy, Susac syndrome and others (Caprara and Grimm, 2012; Gilmour, 2015; Hartnett, 2017; Sigler et al., 2014). In all instances, key features include elevated vascular permeability and edema, which can affect central vision (Ho et al., 2016). Furthermore, sustained retinal ischemia can also result in the formation of new blood vessels that grow toward the vitreous cavity without resolving retinal hypoxia (Fukushima et al., 2011; Ho et al., 2016).

Clinically, ocular levels of VEGF-A and PlGF are elevated in RVO (Aiello et al., 1994; Noma et al., 2015). An analysis of aqueous samples taken from controls and patients with BRVO showed marked elevations of both VEGF-A and PlGF (Noma et al., 2014), and importantly a significant positive correlation for both VEGF-A and PlGF with increasing levels of ischemia (Ryu et al., 2021). These positive correlations are also observed in patients who have CRVO (Noma et al., 2015). Furthermore, significant correlations were observed between levels of PlGF and soluble ICAM-1, PDGF-AA, CCL2, and IL-8 (Noma et al., 2014, 2015), which implicates activation of microglia and macrophages by VEGFR1 as part of the disease pathology in RVO patients.

As with DME, current treatment strategies for RVO include removal of the angiogenic and inflammation drivers or the use of anti-VEGFs and steroids to reduce the overall inflammatory response. Intravitreal use of aflibercept and ranibizumab is effective in reducing edema and restoring visual acuity in patients with BRVO (Campochiaro et al., 2010, 2015) and CRVO (Boyer et al., 2012; Brown et al., 2010; Holz et al., 2013). Furthermore, vision gains and maintenance of vision were reported in patients with significant areas of non-perfusion as a result of CRVO in the COPERNICUS and GALILEO studies (Feltgen et al., 2019; Pielen et al., 2017).

Clinical comparative studies show that agents that target VEGF-A only, or those that bind VEGF-A and PlGF, both provide robust responses in improving vision and reducing macular edema. Clinical study outcomes are particularly relevant, as any emerging signals are observed despite the inherent variability between patients. In a comparator trial in DME (Protocol T) (Wells et al., 2015, 2016), statistical superiority in visual acuity gains was shown, and in RVO trials (LEAVO (Hykin et al., 2019); SCORE 2 (Scott et al., 2017)), less frequent treatment was required and fewer non-responders were observed with aflibercept versus bevacizumab* and/or ranibizumab. In contrast, effects on inflammatory readouts (e.g. aqueous CCL2, IL-6, IL-8 and others) appear to be similar (Cui et al., 2021; Kotake et al., 2019). This is different from what was observed in DR and might be due to the different pathogenic contributions of PlGF signaling in RVO versus DR, or to differences in the methodologies used to measure these agents in the aqueous.

The pathogenesis of RVO and its subtypes has been described in clinicopathological studies (Green et al., 1981; Powner et al., 2016; Wolter, 1961), but its pathophysiology is less clear. Nevertheless, attempts have been made to further explore potential pathobiological mechanisms of RVO in animal models, typically using laser photocoagulation. Histological studies on primate RVO models conducted in the 1970s have described the time course of degenerative changes of the vessels and the surrounding tissue after occlusion (Hockley et al., 1976, 1979), which match human histology. In addition, retinal vessel occlusion models have been generated and explored in many different animals, usually with a focus on the vascular and edematous changes and retinal atrophy (Khayat et al., 2017). Transcriptional profiling in rabbits and mice has shown a strong upregulation of genes associated with hypoxia, angiogenesis, cell damage and inflammation (Martin et al., 2018; Neo et al., 2020) after occlusion. In a non-human primate RVO model, ranibizumab attenuated retinal edema and atrophy but did not affect expression of CCL2, IL-6 and angiopoietin-1/2 (Inagaki et al., 2020), reflecting the clinical findings mentioned above. On a cellular level, activation of microglia and invasion of macrophages from the systemic circulation are prominent responses to experimental BRVO in mice (Ebneter et al., 2017). Remarkably, the invading macrophages seem to have a protective effect on the vein ECs (VanderVeen and Cataltepe, 2019), but whether VEGFR1 signaling is relevant upstream or downstream of the inflammatory response, specifically in RVO, is not known.

### 4.3. Retinopathy of prematurity

ROP is a vasoproliferative disorder of premature infants born with an incompletely vascularized retina. A mismatch between oxygen levels of the in utero and postnatal environments (exacerbated by supplemental oxygen), can lead to delayed retinal vascularization, hypoxia and excessive angiogenic stimuli in the peripheral retina. This results in abnormal vessel growth, in particular at the leading edge of the developing retinal vasculature, and can cause loss of vision through macular dragging and tractional retinal detachment (Mintz-Hittner et al., 2011). Clinically, ROP is classified according to the extent of retinal vasculature development (zone I is the smallest, most central region, and zone III the largest). Also relevant are the circumferential extent (described using hours of a clock face), the severity (stage 1–5, with 5 being the most severe) and the presence of dilated, tortuous posterior pole vessels (referred to as ‘plus’ disease) (Agarwal and Jalali, 2018; International Committee for the Classification of Retinopathy of Prematurity, 2005).

The standard of care treatment for ROP is laser ablation of the peripheral - not yet vascularized - retina, removing the source of the excessive angiogenic stimulus. Alternatively, anti-VEGFs can be used to directly counteract the main angiogenic mediator in the retina, but this approach is still novel and its place in the therapeutic armamentarium remains to be established (VanderVeen and Cataltepe, 2019). The BEAT-ROP study was the first large randomized trial and demonstrated a superiority of bevacizumab* to laser treatment in recurrence rate and unfavorable outcome in zone I eyes, with the caveat that this was not the case for zone II eyes and recurrence after laser treatment was unusually high in this study (Mintz-Hittner et al., 2011; VanderVeen and Cataltepe, 2019). Nevertheless, in the following years several smaller case series studies showed positive effects of bevacizumab* and ranibizumab (Moran et al., 2014; Stahl et al., 2018, 2019; Yang et al., 2018; Yoon et al., 2017). Similarly, aflibercept has also been shown to be effective in ROP (Salman and Said, 2015), and further clinical studies are underway assessing aflibercept versus laser treatment in ROP (e.g. NCT04004208 [FIREFLEYE]). However, treatment decisions, such as laser versus anti-VEGFs or the optimal timepoint for anti-VEGF injection, depend on multiple variables and are still not straightforward (Stahl, 2018).

The effects of intravitreally injected anti-VEGFs can be clinically observed within a few days, when retinal vessels start to grow into the avascular peripheral retina. This apparently paradoxical, pro-angiogenic effect of an anti-angiogenic drug can be explained by a normalization of excessively high VEGF levels, which are known to prevent normal vascular development from studies in model systems (Bentley et al., 2009; Gerhardt, 2008). However, correct dosage is clearly a crucial issue here as excess inhibition of VEGF-A can prevent retinal vascularization as it has been shown in a canine model of ROP (Lutty et al., 2011). To what degree VEGFR1 signaling might be relevant in ROP is however less obvious. A retrospective study (single-center) compared ranibizumab versus aflibercept and found that the need for retreatment was lower and recurrences were delayed in the aflibercept group (Sukgen and Kocluk, 2019). This hints towards an involvement of PlGF or VEGFR1 in ROP but more clinical studies are needed to draw firmer conclusions.

Nevertheless, some insights about the potential functions of VEGFR1 and PlGF in ROP have been gained from animal models. The aberrant angiogenesis that develops in ischemic retinas can be mimicked in neonatal mice or rats by exposing them temporarily to high atmospheric oxygen, which leads to premature cessation of vascularization followed by an increase in retinal VEGF-A levels when the animals are returned to room air and the development of oxygen-induced retinopathy (OIR) (Kim et al., 2016; Smith et al., 1994). PlGF levels have also been shown to increase in OIR rodent models (Ozgurtas et al., 2016; Sato et al., 2009). Notably, the formation of neovascular tufts is suppressed by deletion of the *Pgf* gene or administration of an anti-VEGFR1 antibody in mouse OIR (Carmeliet et al., 2001; Luttun et al., 2002). Furthermore, in comparison to a VEGFR2 specific antibody, the anti-VEGFR1 antibody was equally effective at reducing neovascularization and even more effective at preventing BRB breakdown (Huang et al., 2011). It is unlikely that the effects of the anti-VEGFR1 antibody are based on preventing VEGF-A from binding to VEGFR1 (i.e. inhibiting the endogenous VEGF-A inhibitor) and raising VEGF-A levels, because it is established that rising VEGF-A levels in the OIR model worsen neovascularization. It can therefore be assumed that the relevant mechanism here is the blocking of direct VEGFR1 signaling, affecting either vascular leakage or inflammation. As discussed in sections 3.2 and 3.3, there is evidence for both scenarios.

It is well known that the neovascular response in the OIR model is accompanied by upregulation of inflammatory signals, infiltration of mononuclear phagocytes and activation of resident microglia (Binet et al., 2020; Brockmann et al., 2018; Davies et al., 2006; Sun et al., 2017; Wang et al., 2020). This relates to preterm human infants where the risk of ROP is associated with sepsis (Huang et al., 2019b) and systemic inflammation (Lee and Dammann, 2012; Sood et al., 2010). More specifically, soluble VEGFR1, IL-8, TNF-α and other inflammation-associated proteins in the serum were found to be associated with increased risk in early ROP (Holm et al., 2017).

Experiments in the OIR model have shown that elimination of mononuclear phagocytes leads to decreased neovascular tufts and facilitates vascular regeneration, demonstrating a functional role (Kubota et al., 2009). The contribution of mononuclear phagocytes towards aberrant angiogenesis is however unlikely to be mediated via VEGF-A, because macrophage-specific deletion of VEGF-A, HIF-1A, or EPAS1 had no impact on VEGF-A levels in whole retinas, or on neovascularization in the OIR model (Liyanage et al., 2016; Nürnberg et al., 2018). Nevertheless, mononuclear phagocytes might contribute indirectly to VEGF-A upregulation via activation of Müller glia in ischemic retinas (Nürnberg et al., 2018). In this setting, the specific functions of the VEGFR1 signaling in mononuclear phagocytes await elucidation.

## 5. Role of VEGFR1 in age-related macular degeneration

The relationship between the eye and the immune system has often been considered one of “immune privilege,” in which a combination of the physical BRB and an inhibitory ocular microenvironment (such as high levels of TGFβ) serve to limit local immune and inflammatory responses in order to preserve vision (Zhou and Caspi, 2010). However, accumulating evidence suggests a role for chronic inflammation in the pathogenesis of retinal diseases, including AMD and DR (Chen and Xu, 2015; Guillonneau et al., 2017; Whitcup et al., 2013). So that we may further explore the role of VEGFR1 on the migration and localization of inflammatory cells within the posterior eye with macular degeneration, we should first fully understand those processes, schematically represented in Fig. 1.

In advanced disease conditions such as GA, in which there has been RPE death and the photoreceptor cell layer shows signs of degeneration (Fleckenstein et al., 2018; Sarks, 1976), a substantial body of evidence implicates the subretinal infiltration and accumulation of mononuclear phagocytes (Combadiere et al., 2007; Eandi et al., 2016; Gupta et al., 2003; Hu et al., 2015; Lad et al., 2015; Lavalette et al., 2011; Levy et al., 2015a, 2015b; Penfold et al., 2001; Sennlaub et al., 2013). Mononuclear phagocytes also play a critical role in photoreceptor degeneration and the recruitment and activation of inflammatory cells is thought to exacerbate photoreceptor cell death in retinal degenerative conditions such as AMD (Akhtar-Schafer et al., 2018; Combadiere et al., 2007; Cruz-Guilloty et al., 2013; Guillonneau et al., 2017; Kohno et al., 2013; Rutar et al., 2012; Sennlaub et al., 2013; Suzuki et al., 2012). This is supported by findings from Bhutto et al. who showed that donor eyes with AMD show increased numbers and degranulation of mast cells. It is speculated that mast cell degranulation may, through release of proteolytic enzymes, contribute to death of the choriocapillaris and the RPE and ultimately to CNV formation (Bhutto et al., 2016).

Deposits of soft drusen – lipoproteinaceous debris – within Bruch’s membrane and adjacent to the RPE are a known hallmark of early and intermediate AMD (Fleckenstein et al., 2018; Guillonneau et al., 2017; Sarks, 1976), and represent a known risk factor for progression to advanced or late-stage AMD (Klein et al., 2004). More recently, reticular drusen, observed as discrete yellow-white subretinal dots on fundoscopy, have also been implicated in late AMD, as they appear to affect photoreceptor integrity and are associated with RPE damage (Greferath et al., 2016). Interestingly, in late-stage AMD, presence of reticular drusen is also associated with significantly thinner choroids (Cheung et al., 2018a; Thorell et al., 2015), offering additional insights in the interplay of choroidal and retinal inflammatory processes as AMD progresses. Both large classical drusen and reticular drusen are characterized by the accumulation of mononuclear phagocytes in the subretinal space (Combadiere et al., 2007; Eandi et al., 2016; Greferath et al., 2016; Guillonneau et al., 2017; Levy et al., 2015a; Sennlaub et al., 2013).

Mononuclear phagocytes have been identified in donor tissues using various markers: *Ricinus communis* agglutinin-I (Gupta et al., 2003), C-X3-C motif chemokine receptor 1 (CX3CR1) (Combadiere et al., 2007), CD18 (Combadiere et al., 2007; Levy et al., 2015a; Sennlaub et al., 2013), Iba1 (Sennlaub et al., 2013), CD163 (Lad et al., 2015), and CD14 (Eandi et al., 2016). Although CX3CR1, CD18, and Iba1 are expressed on ramified microglial cells, the presence of CD163-positive and CD14-positive mononuclear phagocytes in AMD demonstrates an activation of microglial cells and/or the recruitment of monocyte-derived macrophages, supporting the notion that AMD may be an inflammatory disease. Interestingly, a significant portion of subretinal mononuclear phagocytes in GA, as well as in and around large drusen, express CCR2, the receptor for the chemokine CCL2, which is only expressed on inflammatory monocytes and early monocyte-derived macrophages (Sennlaub et al., 2013), indicating that the subretinal infiltrate comprises a mixture of monocyte-derived macrophages and activated microglial cells. McLeod et al. (2016) describe significantly increased numbers of Iba1-positive macrophages in the choroid of eyes with signs of early and intermediate AMD. In addition, numbers of HLA-DR-positive submacular macrophages were significantly increased in all stages of AMD, and they exhibited morphologic features suggesting an activated state (McLeod et al., 2016). Debate remains regarding how best to distinguish resident and infiltrating mononuclear phagocyte populations within the retina or the choroid. A subset of mononuclear phagocytes may be clinically visible in AMD patients and a significant number of mononuclear phagocytes (identified using immunohistological markers) in AMD donor eyes contain melanosomes, presumably from ingested RPE debris (Lad et al., 2015; Sennlaub et al., 2013).

The presence of hyperreflective foci is related to neovascular AMD severity (Altay et al., 2016). A prospective, observational study conducted by the Age-Related Eye Disease Study 2 group found that proliferation and inner retinal migration of hyperreflective foci detected by spectral-domain OCT were correlated with the progression of AMD disease in terms of RPE atrophy and expansion of GA (Christenbury et al., 2013; Leuschen et al., 2013). One could postulate that in addition to migrating RPE cells, these cells could be melanin-containing mononuclear phagocytes (Zanzottera et al., 2015). Hyperreflective foci are reduced following anti-VEGF treatment; however, the heterogeneity of the anti-VEGF treatment effects, as well as the mechanism behind this phenomenon, are currently unclear (Coscas et al., 2013; Ota et al., 2010; Sennlaub et al., 2013). Thus, although it has been confirmed that relevant inflammatory cells are implicated in the pathogenesis of AMD disease, these prior studies did not explicitly examine whether VEGFR1 was expressed in these cells. Consequently, we must turn to preclinical studies to assess the role VEGFR1 may play.

Despite major differences between the murine model and human disease processes, laser-induced CNV in the mouse model is used extensively in retinal research as it does mimic the main phenotypical features of exudative AMD (Akhtar-Schafer et al., 2018; Tsutsumi et al., 2003). The laser ruptures the RPE layer and Bruch’s membrane causing a rapid recruitment of mononuclear phagocytes, and, within a few days, choroidal capillaries penetrate into the retina (Akhtar-Schafer et al., 2018). The laser CNV model has been used to demonstrate that blocking inflammatory macrophage recruitment strongly reduces CNV (Akhtar-Schafer et al., 2018; Tsutsumi et al., 2003). The role of VEGFR1 itself was recently shown in the development of CNV post-laser treatment using a tetrameric tripeptide, iVR1, directed against the receptor and specifically developed as an antagonist of VEGFR1 (Tarallo et al., 2020). Intravitreal administration of iVR1 potently inhibited laser-induced CNV in a dose-dependent manner seven days after laser-included damage (reduction of CNV volume by around 70%, p = 0.0002 compared with DMSO control injections). Remarkably, this treatment was more effective than an anti-mouse VEGF-A polyclonal antibody (which achieved a reduction of CNV volumes of around 50%, p = 0.001). The observation points to the involvement of VEGFR1 in pathological neovascularization and therefore blocking this receptor provides a potential alternate treatment route besides VEGF blockade. Interestingly, when a chemically slightly modified version of iVR1 was administered by gavage, a significant reduction in CNV was also observed (around 50% of CNV volume, p = 0.001).

Macrophage-derived VEGF-A does not contribute significantly to CNV development in mice (Huang et al., 2013; Liyanage et al., 2016). However, VEGF-A can promote mononuclear phagocyte recruitment and infiltration in models of laser-induced CNV (Balser et al., 2019). This effect could be due, in part, to the stabilizing effect of VEGF inhibition on the local vasculature as well as to a direct effect of VEGF-A on mononuclear phagocytes. Indeed, circulating human monocytes, which participate in mononuclear phagocyte infiltration in AMD, express VEGFR1 but not VEGFR2, and a VEGFR1-neutralizing monoclonal antibody has been shown to significantly suppress VEGF-A-induced migration of microglial cells (Sawano et al., 2001). In addition, Massena et al. described a specific population of neutrophils, characterized as CD49d^+^CXCR4^high^VEGFR1^high^, that migrate to hypoxic areas, potentially enhancing angiogenesis (Massena et al., 2015). This process is dependent on neutrophil VEGFR1 and endothelial VEGFR2 expression. Recruited neutrophils co-express CD49d, CXCR4, and VEGFR1 and use VLA-4 integrin to facilitate extravasation: VEGFR2 is not expressed by this specific, pro-angiogenic population (Massena et al., 2015).

Similarly, a comparison of an RNA sequencing database (Immunological Genome Project, immgen.org) revealed that monocytes, but not microglial cells, express significant amounts of VEGFR1, while neither cell expresses VEGFR2 in this analysis, at least in mice. Consistent with the role of VEGFR1 in monocyte recruitment to the laser-injured subretinal space is the observation that the blockade of PlGF (Crespo-Garcia et al., 2017) and VEGFR1, but not VEGFR2, inhibits the peak of mononuclear phagocyte recruitment three to four days following laser injury (Huang et al., 2013). The inhibitory effect of VEGFR2-blockade at 14 days after laser impact (Huang et al., 2013) is likely due to the direct effect of VEGF-A/VEGFR2 on vascular permeability.

**Fig. 5.**
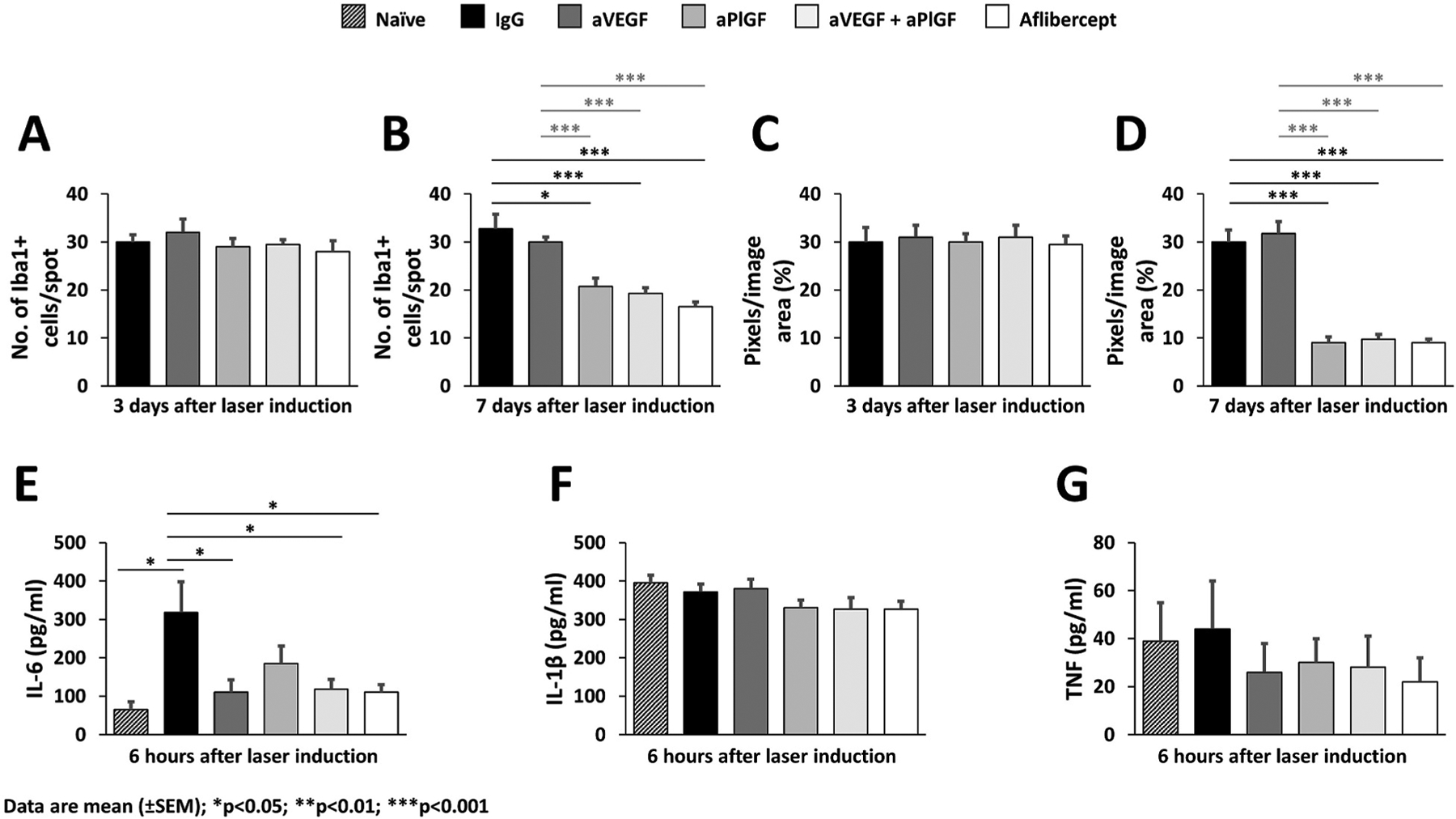
Effects of PlGF and VEGF inhibition on mononuclear phagocytes in retinal flat mounts in the laser-induced mouse model of CNV. **A and B.** Quantification of microglia/macrophages per laser spot in retinal flat mounts 3 and 7 days, respectively, after laser-induced damage. **C and D.** Quantification of ionized calcium-binding adaptor molecule 1 signals 3 and 7 days, respectively, after laser coagulation in retinal flat mounts by counting the mean of colored pixels per image. **E, F, and G.** Interleukin-6, interleukin-1β, and tumor necrosis factor levels, respectively, in retinal flat mounts 6 h after laser damage quantified by enzyme-linked immunosorbent assay with naive (not lasered) animals used as controls. Reproduced under Creative Common CC-BY license (Balser et al., 2019).

Two recent studies investigated the role of PlGF and VEGF-A inhibition on neovessel formation and mononuclear phagocyte reactivity in the murine laser-CNV model (Balser et al., 2019; Crespo-Garcia et al., 2017). Both reports showed that PlGF inhibition, particularly with aflibercept, dampened vascular leakage and CNV. Importantly, blocking PlGF and VEGF-A, but not VEGF-A alone, prevented the accumulation of reactive microglia and macrophages in the lesion area. Higher levels of PlGF and VEGF-A were detected in the laser-damaged retina by immunostaining, and in situ co-expression of PlGF and VEGF-A was demonstrated by the presence of Iba1-positive mononuclear phagocytes in the RPE/choroid complex. These data were verified by quantitative enzyme-linked immunosorbent assays, again demonstrating a strong induction of VEGF-A and PlGF protein levels in the laser-CNV model and effective inhibition of both factors, especially with aflibercept (Fig. 5) (Balser et al., 2019).

Use of intravitreally administered anti-VEGF agents has been associated with a reduction in intraocular (aqueous humor/vitreous humor) levels of selected pro-inflammatory cytokines that are produced by mononuclear phagocytes (Noma et al., 2017), which could indicate that inhibiting VEGF reduces the inflammation in human patients. AMD patients treated with intravitreal ranibizumab or aflibercept displayed a reduction in aqueous humor levels of inflammatory factors and VEGF-A, which accompanied improvements in visual acuity and central macular thickness (Motohashi et al., 2017). Furthermore, some studies have shown increased growth factors and pro-inflammatory mediators after anti-VEGF treatment in subpopulations of patients categorized as low- to non-responders (Pongsachareonnont et al., 2018), suggesting that there is complexity in the underlying mechanisms of inflammation, and in the identification and role of relevant mononuclear phagocytes.

In summary, these reports suggest that the main effect of VEGF-A and PlGF on subretinal inflammation is the participation of VEGFR1 signaling in monocyte recruitment and activation of retinal mononuclear phagocytes. In human AMD, indirect evidence suggests that a similar VEGF-A/PlGF-mediated mechanism might contribute to the pathogenic retinal inflammation. Aqueous humor concentrations of CCL2, which is expressed by subretinal mononuclear phagocytes in AMD (Grossniklaus et al., 2002; Sennlaub et al., 2013) and implicated in inflammatory monocyte recruitment, is reduced following two months of anti-VEGF therapy (Motohashi et al., 2017). In addition, anti-VEGF-A/anti-PlGF therapy reduces the number of hyperreflective foci that likely represent pigment-laden infiltrating mononuclear phagocytes, at least in part (Coscas et al., 2013). Taken together, the inhibition of VEGFR1 signaling might contribute to the beneficial effects of anti-VEGF-A/anti-PlGF in AMD, as it is likely to help control the pathogenic inflammation.

Results of a number of randomized clinical studies comparing anti-VEGF-A therapies with the anti-VEGF-A/anti-PlGF therapy have been published, but it remains unclear whether there are distinct differences between therapies in terms of vision improvements or anatomical benefits. One recent meta-analysis of observational studies with wet AMD patients suggests that an anti-VEGF-A/anti-PlGF strategy may be more beneficial for those patients with initial reduced vision (Zhang et al., 2017), which would likely be a more difficult-to-treat population. This would suggest a potential benefit of VEGFR1 inhibition by agents that block all VEGFR1 ligands including PlGF, such as aflibercept and conbercept*, versus other anti-VEGFs such as ranibizumab, bevacizumab* or brolucizumab, which do not bind PlGF. It has been established that inflammation and hypoxia are important contributors to retinal vascular and choroidal diseases. Notably, inflammatory cells such as microglia and monocytes upregulate VEGFR1, and it has also been shown that VEGFR1 is the sole VEGF receptor upregulated during hypoxic conditions (Miyamoto et al., 2007). There is a clear need in this area to better understand, from the clinical perspective, the interplay between microinflammation and angiogenesis in CNV. Furthermore, studies that examine the interplay of target binding affinity and potency, intraocular half-life and duration of VEGF suppression within the eye may provide further context to assign relative order of importance for understanding clinical differences in efficacy among the agents.

## 6. Future directions

To date, the use of VEGF inhibitors has revolutionized the treatment of retinal diseases characterized by neovascularization, including AMD and DR, with many of these agents coming to represent new standards of care (Bakri et al., 2019; Framme et al., 2018; Singh et al., 2019; Tsilimbaris et al., 2016). Estimates suggest that more than 90% of patients with AMD treated with anti-VEGF therapy avoid moderate to severe vision loss (Miller, 2016). However, despite these therapeutic successes, a subset of patients fails to respond to anti-VEGF therapy or show sub-optimal or a diminishing response over time (Nguyen et al., 2018). It is therefore important to understand the mechanistic basis of these shortcomings so we can continue to raise therapeutic standards. As our understanding of the pathogenesis of ocular neovascular diseases continues to evolve and potential new therapeutic targets and/or formats of anti-VEGF mediated mechanisms of action are being identified, the hope is that this progress can be translated into the clinical setting (Table 1).

### 6.1. Receptor–ligand interrelationships and localization

One of the biggest challenges in this field remains the lack of clarity around the functional relevance of ligand and receptor heterodimers versus homodimers. As discussed in this review, it is possible for heterodimers to have stimulatory as well as inhibitory effects depending on the circumstances. Unless we fully understand the full range of relevant molecular interactions between VEGF-A, VEGF-B and PlGF, we will continue to struggle with the interpretation of experimental manipulations and clinical observations. Furthermore, it is essential to gain a clearer picture about which cells send signals and which cells receive them, including within inflammatory cell subpopulations, and further experimental research is clearly needed here. In this context it is important to keep in mind that there may be important differences between animal models and humans with regards to the prevalence of isoforms and heterodimers in the VEGF ligand/receptor family as well as immune cell subpopulations. It is likely that the rapidly evolving technologies enabling integrative single-cell analysis (Stuart and Satija, 2019) will be transformational in this field.

### 6.2. Research models

We have discussed the complexity of the pathogenic processes in this review and identified some of the multifactorial interactions between different cell types. An ongoing need exists for new, or modified, research models with which to explore those cellular interactions and their impact on neovascular disease and retinal and choroidal vascular permeability disorders. Although existing preclinical models have proven helpful – and new models continue to emerge (Kitahara et al., 2018; Morita et al., 2018) – they never fully represent a given human pathology. An overreliance on animal models that phenocopy particular traits of human eye diseases (e.g. neovascularization) may arguably be at least in part responsible for the high failure rate at the transition from preclinical proof of concept to clinical application. Instead, models that aim to improve our understanding of specific molecular interactions and signaling pathways, which have been validated as relevant in humans, are more likely to advance therapeutic development.

### 6.3. Inflammation

We have also seen that questions remain regarding VEGFR1 in the context of inflammatory cells in ocular disease processes. These concern the potential differentiating effects of blocking PlGF versus VEGF-A or VEGF-B on inflammatory processes; the specific role of VEGFR1 on microglia/macrophages (Ding et al., 2018) in terms of downstream signaling; the possible role of co-receptors; the composition and dynamics of different immune cell subpopulations; and the consequences of selective targeting with pharmacological agents. Recent human data have implicated inflammatory processes in AMD and DR, with high intraocular levels of VEGF family ligands (e.g. VEGF-A and PlGF) and pro-inflammatory cytokines (e.g. TNF-α and CCL2) reported (Ten Berge et al., 2019; Tsai et al., 2018), although the impact of immunomodulation at the cellular level remains unclear. In patients with various retinal diseases, only a limited number of studies have examined the effects of anti-VEGF treatment on aqueous/vitreous levels of selected pro-inflammatory cytokines (Motohashi et al., 2017; Noma et al., 2017). Further robust studies are required to clarify the complex relationships between intravitreal VEGF-A or PlGF inhibition and effects on downstream mediators quantified in aqueous/vitreous media.

## 7. Conclusions

This review has delved into the context and role of VEGFR1 in retinal and choroidal vascular diseases, providing updates on the requisite preclinical studies and clinical context that inform on emerging evidence of potentially greater contributions by VEGFR1 to these mechanisms than were previously recognized. Signal transduction following VEGF-A, VEGF-B, or PlGF binding to relevant VEGFRs was examined in the context of known, versus yet to be elucidated, downstream activity. Preclinical studies have shown that VEGFR1 acts as a decoy receptor, and that transduction signaling following VEGF-A versus PlGF binding to VEGFR1 may demonstrate attenuated versus potentiated signaling. Thus, further work is merited to clarify the differences and the downstream consequences of these differences in retinal diseases.

Advances have been made in the understanding and the importance of VEGF-A/PlGF heterodimers, VEGFR1/VEGFR2 heterodimers versus homodimeric ligands and/or homodimeric VEGF receptors in the context of the pathobiology of retinal diseases and other relevant organ systems. It remains challenging, however, to show clear clinical benefit of specific ligand/receptor interactions in retinal disease function. Greater understanding will require more nuanced studies to interpret and establish robust relationships.

Both preclinical and clinical studies in retinal vascular diseases such as diabetic eye disease, RVO and ROP have provided support and understanding for the contributory role of inflammation mediated by VEGFR1/PlGF/VEGF-A. The results of well-designed comparative trials may provide insights regarding the functional benefit of specific anti-VEGF agents with different target profiles. As discussed in section 4, in retinal vascular diseases such as DME and RVO some trials have shown outcome differences between aflibercept and other agents that bind VEGF-A only, which has led to speculation concerning a potential role for PlGF inhibition contributing to these differences. Further characterization of the molecular features and pharmacokinetic profiles for these agents may contribute to our understanding and support our quest to explain these clinical differences. In choroidal vascular diseases, preclinical evidence is just beginning to clarify a role for VEGFR1/PlGF in their pathobiology (Balser et al., 2019; Crespo-Garcia et al., 2017). Prior mechanistic work in multiple studies has confirmed that mononuclear phagocytes contribute to the pro-inflammatory environment within AMD (Kauppinen et al., 2016; Knickelbein et al., 2015). It remains to be determined whether VEGFR1 is induced in these mononuclear phagocytes which, upon binding to VEGF-A or PlGF, in turn contributes to the inflammatory milieu described by such studies. Even as experiments have confirmed the major contribution of inflammation to the pathology of AMD, most studies have not examined whether VEGFR1 is induced in the mononuclear phagocytes. Thus, an opportunity remains to better understand the potential interplay between known complement cascades within the retina and VEGFR1-induced inflammatory cytokines, and to further examine and/or establish a role for VEGFR1/PlGF and its clinical relevance in AMD.

## Figures and Tables

**Table 1:** Currently approved and emerging anti-VEGF therapies and ongoing clinical trials.

Drug	Molecular Features	Approval Status	Ongoing Trials
Ranibizumab (LUCENTIS)	Fab against all VEGF-A isotypes	First approval 2006	LUMINOUS – observational study
		Multiple retinal disease indications	Control arms for multiple emerging anti-VEGFs and other targets
Aflibercept (EYLEA)	Fusion protein against all VEGF-A isotypes, VEGF-B and PlGF	First approval 2011	Multiple observational studies
		Multiple retinal disease indications	Control arms for multiple emerging anti-VEGFs and other targets
Brolucizumab (BEOVU)	scFv against all VEGF-A isotypes	First approval 2019 nAMD	Multiple trials initiated/planned in DME, RVO, and T&E trials in nAMD
Conbercept	Fusion protein against all VEGF-A isotypes and PlGF	Approval (China) 2013 nAMD	Phase III
Abicipar	DARPin against all VEGF-A isotypes	Phase III	Phase III development in nAMD (not approved by FDA [June 2020])
Faricimab	Antibody against VEGF-A and anti-Ang2	Phase III	Phase III development in nAMD and DME

Ang2, angiopoietin-2; DARPin, designed ankyrin repeat protein; DME, diabetic macular edema; Fab, monoclonal antibody fragment; nAMD, neovascular age-related macular degeneration; PlGF, placental growth factor; RVO, retinal vein occlusion; scFv, single-chain variable fragment; T&E, treat and extend; VEGF-A, vascular endothelial growth factor-A.

## Footnotes

* Note on drug approval status: Bevacizumab is not approved for intraocular use, even though it is used off-label in several countries; conberecpt is currently only approved and used in China.

## Abbreviations

AMD: Age-related macular degeneration
BRB: Blood–retina barrier
BRVO: Branch retinal vein occlusion
CCL2: CC chemokine ligand 2
CNV: Choroidal neovascularization
CRVO: Central retinal vein occlusion
CX3CR1: C-X3-C motif chemokine receptor 1
DME: Diabetic macular edema
DR: Diabetic retinopathy
EC: Endothelial cell
eNOS: Endothelial nitric oxide synthase
ERK: Extracellular signal-regulated kinase
GA: Geographic atrophy
HIF: Hypoxia-inducible factor
Iba1: Ionized calcium-binding adapter molecule 1
ICAM: Intracellular adhesion molecule
IL: Interleukin
KO: Knock-out
MAPK: Mitogen-activated protein kinase
NPDR: Non-proliferative diabetic retinopathy
NRP: Neuropilin
OCT: Optical coherence tomography
OIR: Oxygen-induced retinopathy
PI3K: Phosphoinositide 3-kinase
PDGF: Platelet-derived growth factor
PDR: Proliferative diabetic retinopathy
*Pgf*: *Placental growth factor (mouse gene)*
PlGF: Placental growth factor (protein)
ROP: Retinopathy of prematurity
RPE: Retinal pigment epithelium
RVO: Retinal vein occlusion
sVEGFR1: Soluble/secreted VEGFR1
TGF: Transforming growth factor
TK: Tyrosine kinase
TNF: Tumor necrosis factor
VEGF: Vascular endothelial growth factor
VEGFR: Vascular endothelial growth factor receptor
